# Experimental and Clinical Biomarkers for Progressive Evaluation of Neuropathology and Therapeutic Interventions for Acute and Chronic Neurological Disorders

**DOI:** 10.3390/ijms231911734

**Published:** 2022-10-03

**Authors:** Doodipala Samba Reddy, Hasara Nethma Abeygunaratne

**Affiliations:** 1 Department of Neuroscience and Experimental Therapeutics, College of Medicine, Texas A&M University Health Science Center, Bryan, TX 77807, USA; 2 Institute of Pharmacology and Neurotherapeutics, College of Medicine, Texas A&M University Health Science Center, Bryan, TX 77807, USA; 3 Intercollegiate School of Engineering Medicine, Texas A&M University, Houston, TX 77030, USA; 4 Department of Biomedical Engineering, College of Engineering, Texas A&M University, College Station, TX 77843, USA; 5 Department of Veterinary Integrative Biosciences, College of Veterinary Medicine & Biomedical Sciences, Texas A&M University, College Station, TX 77843, USA

**Keywords:** Alzheimer’s disease, biomarkers, brain injury, dementia, epilepsy, neurology, Parkinson’s disease, pathophysiological, inflammation, neurogenesis, neurodegeneration, stroke

## Abstract

This article describes commonly used experimental and clinical biomarkers of neuronal injury and neurodegeneration for the evaluation of neuropathology and monitoring of therapeutic interventions. Biomarkers are vital for diagnostics of brain disease and therapeutic monitoring. A biomarker can be objectively measured and evaluated as a proxy indicator for the pathophysiological process or response to therapeutic interventions. There are complex hurdles in understanding the molecular pathophysiology of neurological disorders and the ability to diagnose them at initial stages. Novel biomarkers for neurological diseases may surpass these issues, especially for early identification of disease risk. Validated biomarkers can measure the severity and progression of both acute neuronal injury and chronic neurological diseases such as epilepsy, migraine, Alzheimer’s disease, Parkinson’s disease, Huntington’s disease, traumatic brain injury, amyotrophic lateral sclerosis, multiple sclerosis, and other brain diseases. Biomarkers are deployed to study progression and response to treatment, including noninvasive imaging tools for both acute and chronic brain conditions. Neuronal biomarkers are classified into four core subtypes: blood-based, immunohistochemical-based, neuroimaging-based, and electrophysiological biomarkers. Neuronal conditions have progressive stages, such as acute injury, inflammation, neurodegeneration, and neurogenesis, which can serve as indices of pathological status. Biomarkers are critical for the targeted identification of specific molecules, cells, tissues, or proteins that dramatically alter throughout the progression of brain conditions. There has been tremendous progress with biomarkers in acute conditions and chronic diseases affecting the central nervous system.

## 1. Introduction

This article describes a comprehensive landscape of major neurological biomarkers of brain disorders including acute neuronal injury, neurodegeneration, neurogenesis, and neuroinflammation. It highlights critical aspects of monitoring the pathophysiology and therapeutic interventions using the most common biomarkers in acute and chronic brain disorder research. It focuses on three core subtypes: blood-based, immunohistochemical-based, and neuroimaging-based biomarkers. Other biomarkers include CSF-based, neurophysiological, and electrophysiological biomarkers. This review addresses such topics as the landscape of biomarker classes, the impact biomarkers can have on various phases of brain research, and key questions and potential opportunities to help advance the implementation of validated biomarkers for neurological disorders. It describes clinical needs, challenges, and potential opportunities to advance the implementation of validated biomarkers for neurological research and clinical diagnostics of neurological conditions. 

The brain is the most complex organ in the human body. Unlike other organ systems, it is extremely difficult to understand the overarching function of brain regions at multiple levels. Tremendous progress has been made in our understanding of the nervous system using advanced tools and technologies. With this information, researchers are gaining new insights into the network function of neurons and multiple interactions between different brain cells (Table 1). Electrophysiological data, functional neuroimaging, and biomarkers provide a systematic understanding of normal brain function, as well as the etiology and pathology of acute and chronic brain diseases.

Approximately 1 billion people worldwide suffer from neurological disorders with progressive loss of neurological functions. They include dementia, stroke, epilepsy, migraines, brain injuries, cancer, and neuroinfections. Neurodegenerative disorders include Alzheimer’s disease (AD), tremor-associated Parkinson’s disease (PD), amyotrophic lateral sclerosis (ALS), Huntington’s disease (HD), and multiple sclerosis (MS). Chronic neurological disorders include epilepsy, migraine, neuropathy, myasthenia gravis, spasticity, and certain genetic disorders. Neuropsychiatric disorders include major depression, anxiety, schizophrenia, bipolar mania, alcohol addiction, and drug abuse.

Neuronal injury and neurodegeneration are the hallmark pathologies in acute brain injury and chronic neurological conditions. Acute neuronal injuries are often fatal conditions such as traumatic brain injury (TBI), ischemic stroke, seizures, and brain infections. Chronic neurodegenerative disorders are devastating and cause disabling ailments, including epilepsy, AD, PD, HD, MS, and ALS. These incurable and debilitating conditions are caused by the chronic and progressive death of neurons in discrete regions of the brain. The inconvenience of obtaining biopsies of the brain limits the definite diagnosis of these diseases [1]. Thus, biomarkers are used for accurate diagnosis and prognosis of diseases and to track the rate of progression. Biomarkers are particularly important indicators for detecting different stages of a disease such as prevention, early onset, treatment, and progression [2].

A biomarker is defined as characteristic that is measured as an indicator of normal biological processes, pathogenic processes, or responses to an exposure or intervention. Biomarkers are biological molecules that define and quantify the characteristics of a biological process effectively [3]. Biomarkers are commonly used in clinical and basic research to provide crucial information on molecular mechanisms, disease detection, pathological processes, and pharmacological responses, which gives rise to therapeutic approaches [3]. They are considered an effective therapeutic tool in facilitating drug development and treatment monitoring. Thus, they act as a frontier for drug discovery and development. Biomarkers can be categorized into four main groups: detective, diagnostic, prognostic, and predictive. The World Health Organization (WHO) defines biomarkers as, “any substance, structure, or process that can be measured in the body or its products and influence or predict the incidence of outcome or disease”. The ideal biomarker should be easily measurable, reproducible, inexpensive, consistent between different populations, and unaffected by external factors [4]. These characteristics of biomarkers can also serve as limitations. Biomarkers are widely used in many areas of research including cancer, toxicology, neurology, etc. DNA-based biomarkers are the most prominently used to detect familiar forms of neurodegenerative diseases, whereas RNA-based biomarkers are a newer approach in neuroscience research [1].

In neurological research, biomarkers are used as a powerful approach for deepening the understanding of mechanisms related to brain disorders and detecting the early onset of a disease [5]. This is due to the limitations of standard staining and biopsy techniques, which require more time and effort to provide a substantial prognosis. For example, inflammation is one of the most common occurrences in neurodegenerative diseases, and the identification of different molecules involved in this process could be used as an easy target for biomarker therapies [4]. Biomarkers are capable of both predicting the onset of disease symptoms and the rate of progression of a disease. This will be extremely useful in predicting how advanced stages of the disease have evolved, specifically in AD, PD, and HD [3]. Because there are distinct stages of severity based on symptom progression, a single biomarker cannot give an accurate assessment of disease progression. Rather, an array of biomarkers is used to validate the seriousness of neurodegenerative diseases [6]. Table 2 shows a list of potential biomarkers currently being used in acute neuronal injuries and neurological disorders.

Biomarkers have been a great discovery for the diagnostic criteria to identify a neurodegenerative disease. This minimizes invasive procedures such as lumbar punctures. Table 3 summarizes the potential experimental and clinical biomarker candidates for neuronal injury and neurodegeneration.

## 2. Immunohistochemical Biomarkers of Neuronal Injury and Neurodegeneration

Immunohistochemistry (IHC) is an effective tool in determining neuropathological changes in various diseases, thereby leading to the identification of novel therapeutic treatments. It is a technique that detects the expression and localization of specific antigens (i.e., proteins) in a tissue sample by using specific antibody–antigen reactions [41]. Table 4 shows a list of reliable potential antibodies used in neuroscience research. The IHC process includes two stages: (1) slide preparation and (2) analysis and quantification of the acquired antigen expression data. The slide preparation phase includes tissue fixation and processing, antigen retrieval, a non-specific binding block, an endogenous peroxidase block, primary antibody incubation, secondary antibody incubation, washing steps, and counterstaining. IHC has remained a standard method for identifying neuronal cell types because it is relatively inexpensive to perform, requires little specialized equipment, employs widely available reagents, and does not damage the morphology of tissues [42].

**Figure 1:**
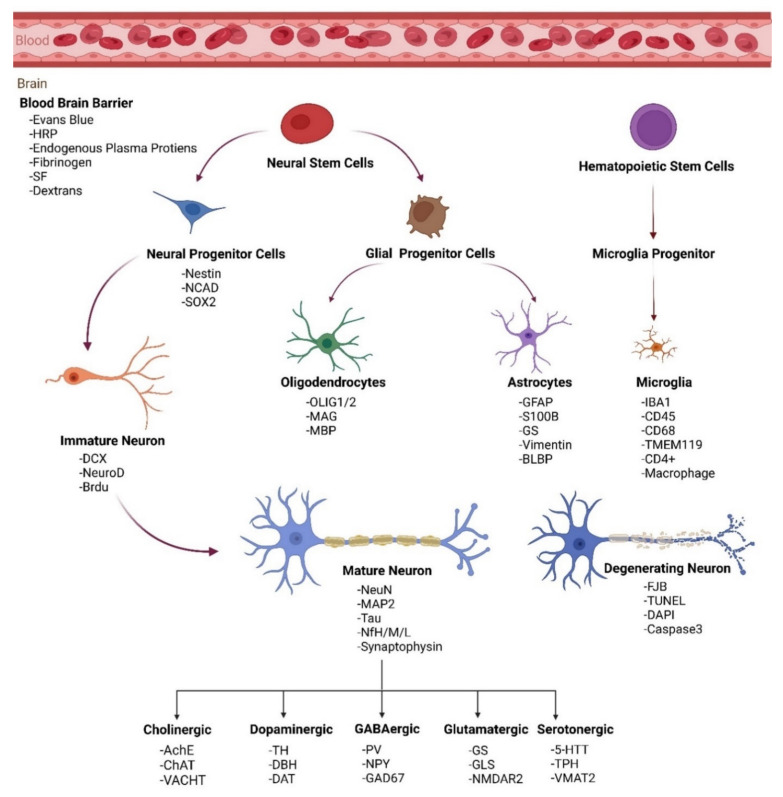
**Cell-type-specific biomarkers for brain disorders**. Schematic representation of neural differentiation and potential neural-specific biomarkers for neurodegeneration and neuronal injury.

Immunohistochemical neuronal biomarkers can be used to measure the protein levels of specific proteins released into the circulation by brain cell injury, blood vessel injury, and inflammation. For example, the neuronal nuclei antigen (NeuN) can be used as a quantitative measure to identify the neuronal loss and number of neurons present in the brain. 4′,6-Diamidino-2-phenylindole (DAPI) can stain the nucleus to distinguish between dying and alive neurons in the brain. Inflammation markers such as the glial fibrillary acidic protein (GFAP) can be used to measure the intensity of inflammation after brain injury or during distinct stages of neurodegenerative diseases [41]. These biomarkers can be used to detect biomechanisms in neurodegeneration and brain injury [51]. IHC can also be used to analyze the disruption to the blood–brain barrier permeability by evaluating endogenous proteins such as albumin, immunoglobulin, and fibrinogen. Neural-specific biomarkers are useful to detect the progression of these neurodegenerative diseases (Figure 1, Table 5). Experiments can be set up to monitor the progressive pathology by creating large initial cohorts of disease models and conducting IHC staining at incremental timepoints [52]. The rapid indication of pathology using this variety of biomarkers will make it possible for more informed patient management and symptomatic treatment.

### 2.1. Neurodegeneration

Neurodegeneration is the progressive loss and dysfunction of neurons in the central nervous system (CNS), which leads to neuronal death. It is a key pathological feature of many acute and chronic neurodegenerative diseases such as PD, AD, stroke, epilepsy, TBI, and MS. The loss of neurons in certain brain subregions can have a devastating influence on the movement, memory, speech, intelligence, etc. of an individual. Hence, it is vital to examine the various aspects of neurodegeneration to assess the disease progression. For this, various histochemical stains and antibodies can be used to detect degenerated and dead neurons. Key neurodegenerative markers used to study the neuropathology are NeuN, parvalbumin (PV), DAPI, Fluoro-Jade B (FJB), and TUNEL. Many neurodegenerative disorders cause changes to the number of neurons in the brain. These changes are often subtle and can only be detected by accurate quantification. 

#### 2.1.1. Counting of Cells in Sections 

In recent years, modern design-based stereology has emerged as a crucial and effective technique for studying the brain. An optimized, unbiased neurostereology can be performed to quantify the number of neurons in different brain regions. The intensity of brain cell injury and neurodegeneration in a variety of neurological models can be analyzed by quantifying the number of total neurons and interneurons in different brain regions by utilizing the optical fractionator formula [141]. Stereology techniques use two-dimensional measurements to provide accurate and quantitative information about a three-dimensional object. Stereology can provide information about relative and absolute cell numbers, cell density, and tissue volume. Software such as NewCast, Cellprofiler, and Stereo Investigator can be used to perform optimized neurostereology quantification.

##### Neuronal Nuclei Antigen Nuclear Protein (NeuN)

**Figure 2:**
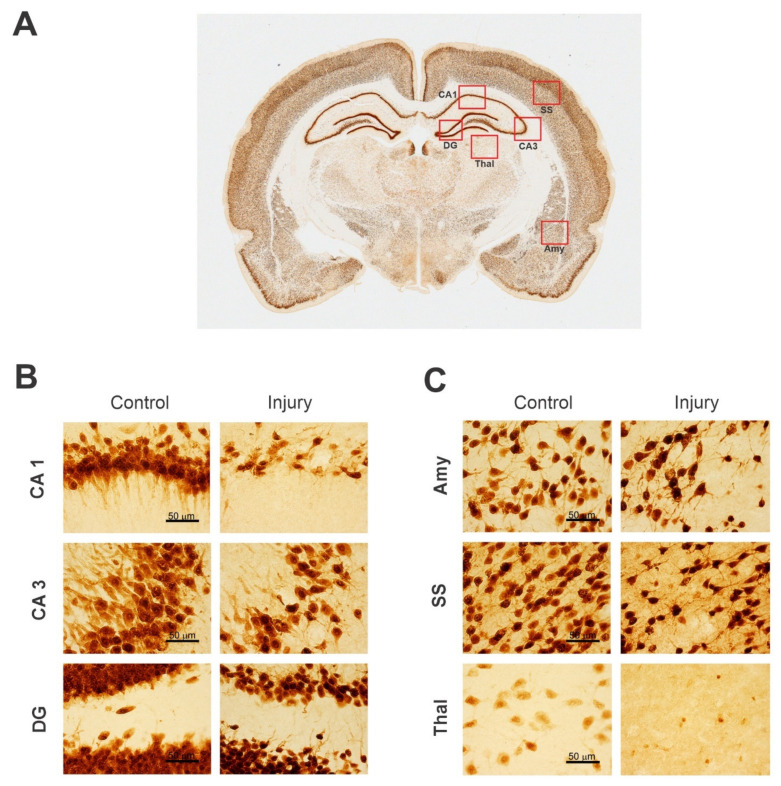
**NeuN(+) immunostaining of principal neurons in the brain**. Representative images of NeuN(+) principal neurons in the brain sections of DFP chemically injured epileptic rats. (**A**) Serial slice of NeuN(+)-stained control rat brain (bregma—1.70 mm), 1.25× objective. The red boxes are regions that are further enlarged in panel B and C. (**B**) NeuN(+) hippocampal subregions: CA1, CA3, and DG of control and epileptic rats, 60× objective. (**C**) NeuN(+) subregions: amygdala (Amy), somatosensory cortex (SS), and thalamus (Thal) of control and epileptic rats, 60× objective. Images were taken via bright-field microscopy. The images were captured based on our lab’s published methodology of DFP-induced neuropathology (Refs. [43,145,146]).

NeuN is an extremely specific protein that is highly expressed in the nucleus and less expressed in the cell body in differentiated neurons of humans and most other vertebrates [142]. NeuN, also named Fox-3/RBFOX3, is found in most neuronal cell types throughout the CNS. NeuN plays a key role in the regulation of alternative mRNA splicing and neural cell differentiation [74]. The NeuN immunohistochemical marker is used to identify neuronal loss due to neurodegeneration and to quantify the total number of neurons in various brain regions. Thus, it is established as a universal marker for neurons. NeuN markers can facilitate the assessment of ganglion cell tumors and hamartomas, hypoxic–ischemic damage, neuronal migration disorders, neurodegenerative diseases, epilepsy, etc. However, a few neuronal cell types are devoid of staining because NeuN is not expressed in glial cells, oligodendrocytes, retinal photoreceptor cells, mitral cells of the olfactory bulbs, and cerebellar Purkinje cells [143]. Another limitation of using NeuN is that it only stains the nuclei of the mature neurons and does not stain the nuclei of immature neurons until they completely grow. A detailed methodology of NeuN histological staining is available in recent papers [43,141,144]. As a representative example, higher-magnification images of NeuN(+)-stained healthy and injured rat brain sections are shown (Figure 2).

##### Parvalbumin (PV)

**Figure 3:**
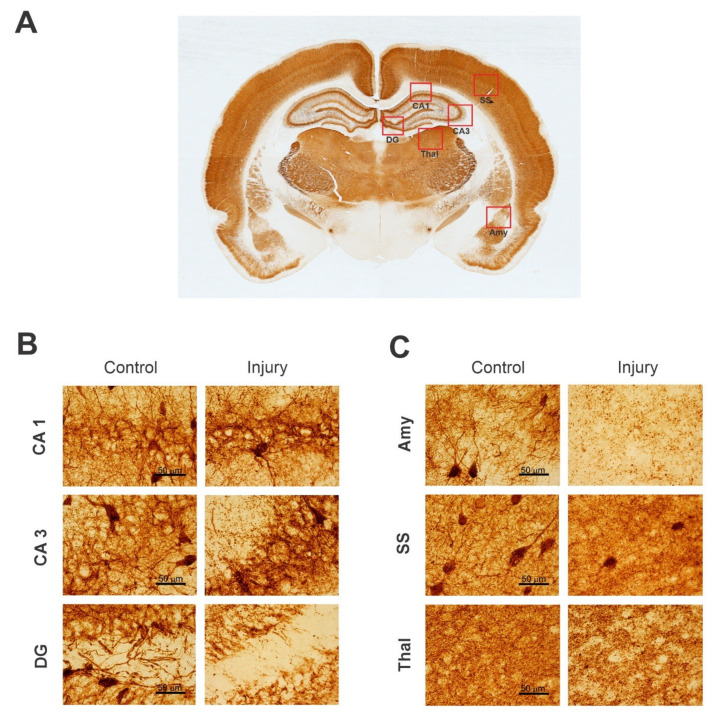
**PV(+) immunostaining of inhibitory interneurons in the brain**. Representative images of PV(+) inhibitory GABAergic interneurons in the brain sections of DFP chemically injured epileptic rats. (**A**) Serial slice of PV(+)-stained control rat brain (bregma—1.70 mm), 1.25× objective. The red boxes are regions that are enlarged in panel B and C. (**B**) PV(+) hippocampal subregions: CA1, CA3, and DG of control and epileptic rats, 60× objective. (**C**) PV(+) subregions: amygdala (Amy), somatosensory cortex (SS), and thalamus (Thal) of control and epileptic rats, 60× objective. Images were taken via bright-field microscopy. The images were captured based on our lab’s published methodology of DFP-induced neuropathology (Refs. [43,145,146]).

PV is a small, stable, calcium-binding albumin protein expressed in inhibitory interneurons [145]. It is localized in both the soma and the neurites of GABAergic neurons [147]. Calcium-binding proteins such as PV play a key role in physiological processes, such as cell-cycle regulation, muscle contraction, organization of microtubules, and photo-transduction. They are important modulators of intracellular calcium dynamics in neurons. Most PV-immunoreactive cells present in the CNS could be considered interneurons and the distribution of PV subpopulations is distinctly found in the rat, cat, and primate cortex [148,149]. PV(+) interneurons belong to the GABAergic family of interneurons that are responsible for modulating the dynamic neuronal responses [150]. PV(+) interneurons can also be divided into two subgroups: basket cells and chandelier cells. These interneurons can be found in muscles, brains, and neuronal hormonal tissue. Most of the PV interneurons are fast spiking. PV fast-spiking interneurons are the most common type of GABAergic interneuron in the neocortex, accounting for roughly 40% of all such neurons [151]. Since the function of PV is reduced in numerous brain diseases, such as epilepsy, understanding which stages and parts of the brain affect the expression of PV can lead to important therapeutic targets [148,150]. Details of immunostaining for PVs has been published previously [144,145]. As a representative example, higher-magnification images of PV(+)-stained healthy and injured rat brain sections are shown (Figure 3).

##### Neuropeptide Y (NPY)

NPY is a 36 amino acid peptide neurotransmitter that is primarily expressed in the peripheral (PNS) and central nervous systems. NPY belongs to a family of neuroendocrine peptides, peptide YY and pancreatic polypeptide. NPY plays many roles in the regulation of brain activity by having a neuroprotective effect, increasing trophic support, controlling calcium homeostasis, and mitigating neuroinflammation. NPY participates in seizure suppression, learning, memory, appetite, and endocrine secretions. These functions are facilitated by at least five different G-protein-coupled receptors (Y1, Y2, Y4, Y5, and Y6) [152,153]. The literature on both human and rodent models has revealed that alterations of NPY levels in the brain are related to several neurodegenerative and neuroimmune diseases such as AD, PD, HD, and Machado–Joseph disease (MJD) [153]. An abnormal increase in NPY expression is correlated to HD and MJD, whereas the decrease in NPY expression has been closely correlated to AD and PD [154]. In the PNS, NPY is expressed in enteric neurons in the adrenal medulla and postganglionic sympathetic neurons [152]. The majority of NPY is expressed in the CNS. It is distributed predominantly in the cerebral cortex, cerebellum, thalamus, hypothalamus, amygdala, hippocampus, and brainstem. The highest amount of NPY is present in the hippocampus. Immunohistochemical studies have revealed that NPY found in the hippocampus, cortex, and amygdala is expressed in GABAergic neurons co-localizing with somatostatin and, to a lesser degree, with PV and nitric oxide [152]. Thus, NPY is used as a biomarker for distinct classes of GABAergic neurons [155]. 

##### Glutamate Decarboxylase 65 (GAD65) and Glutamate Decarboxylase 67 (GAD67) 

Glutamate decarboxylase (GAD) is an enzyme that catalyzes the production of GABA, the main inhibitory neurotransmitter in the mammalian CNS. GABA engages in brain plasticity-related processes, such as learning, memory, locomotion, reproduction, and development of the nervous system [156]. Dysfunction of GABA can result in numerous disorders, including schizophrenia and epilepsy. GAD is expressed as two major isoforms: GAD65 and GAD67, which differ in molecular weight (65 and 67 kDa). GAD65 and GAD67 are encoded by the genes *Gad1* and *Gad2*, respectively. GAD65 is a membrane-anchored protein associated with GABA exocytosis, whereas GAD67 is a cytoplasmic protein associated with GABA metabolism [98]. They are co-expressed in many of the GABA-comprising neurons. There is variety in the distribution of GAD65 and GAD67 within the GABAergic neurons. GAD67 is more abundant in the cell bodies, processes, and synaptic terminals of neurons, whereas GAD65 is highly enriched in axon terminals and is associated with synaptic vesicles [157]. Hence, GAD67 is more often used as an immunohistochemical biomarker for GABAergic brain structures. Both GADs, together with GABA, have provided reliable and specific markers for GABAergic neurons [158]. GAD67 is expressed in neurons of the hippocampus, cortex, cerebellum, olfactory bulb, striatum, paleostriatum, reticular zone of the substantia nigra, and inferior colliculus. Various neurological and psychiatric diseases such as ischemia, schizophrenia, and PD can impair GAD67 expression [157]. The formation of tau-containing extracellular vesicles (EVs), which is a part of AD, is facilitated by GAD67 [159]. This causes an increase in tau phosphorylation and conformational change in recipient neurons. Phosphorylated tau is accumulated in GAD67-catalyzed GABAergic interneurons, contributing to the risk of AD. Most genomic studies show a strong correlation between GAD67 and neurological diseases [160]. 

##### Acetylcholinesterase (AChE)

AChE is a vital enzyme in the nervous system that ceases nerve impulses by catalyzing the breakdown of the neurotransmitter acetylcholine at postsynaptic neuromuscular junctions and brain cholinergic synapses [84]. This terminates continuous nerve firings between synapses. Thus, it is important for the normal functioning of the nervous system. As AChE participates in the cholinergic system function, it is considered a reliable marker for identifying cholinergic neurons [161]. The assessment of AChE inhibition is a widely used biomarker to recognize the effects of pesticides on the nervous system because AChE acts as a specific molecular target of organophosphate and carbamate pesticides [84]. Pathological aging, such as AD, is characterized by neuronal loss, whereas normal aging is characterized by a progressive loss of cholinergic function caused by dendritic, synaptic, and axonal degradation and deterioration in trophic support. AChE plays a key role in cholinergic transmission, which is altered in both the cerebral cortex and the hippocampus due to aging and AD [161]. Hallmarks of AD are commonly related to cerebral β-amyloid (cAβ) accumulation and cholinergic dysfunction [162]. There is no data showing a relationship between cAβ and peripheral AChE changes in the early diagnosis of AD. However, a recent study suggests that there is a decrease in AChE levels in normal individuals with cAβ accumulation compared to those without cAβ accumulation. This could be a potential biomarker for the likelihood of AD [162,163]. 

##### Tyrosine Hydroxylase (TH) 

TH is an enzyme involved in the conversion of the amino acid L-tyrosine to the precursor for dopamine, L-3,4-dihydroxyphenylalanine (L-DOPA). It is the rate-limiting enzyme involved in the synthesis of catecholamines, such as dopamine, noradrenaline, and adrenaline [157]. These neurotransmitters regulate a variety of physiological and psychoemotional functions and reactions, including stress, sleep, learning, memory, attention, and energy metabolism. TH is found distinctly in the cell body and subsidiary in the cell membrane. All four major human TH isoforms are found in neurons [164]. Many neurological and psychiatric disorders, such as PD, AD, schizophrenia, depression, and drug addiction, are pathogenetically influenced by disruptions in TH function. TH is considered a gold standard marker in identifying dopaminergic neurons in the brain, which is specifically relevant for PD research. The TH marker has been used in postmortem studies to quantify the degree of dopaminergic cell loss in Parkinson’s patients [165]. 

#### 2.1.2. Counting Dying Cells in Neurodegeneration

Neuronal cell death is a major pathological hallmark of neurodegenerative diseases. Although it is recognized that neurons die in neurodegenerative diseases, the way cells die is often unclear. There are several recognized ways of neuronal cell death, including apoptosis, necrosis, autophagic cell death (ACD), and excitotoxicity. Apoptosis and necrosis are believed to be the two major modes of neuronal death in neurodegenerative diseases. The fundamental differences between apoptosis and necrosis lie in the different morphologies of the cells and whether the contents of the cells leak out during the process [166]. The role of these cell death pathways in neurodegenerative diseases is unknown, due to the lack of specific markers [167]. The total number of dying neurons in different brain regions can be determined through unbiased stereological analysis and a neuropathology scoring system [145].

##### 4′,6-Diamidino-2-phenylindole (DAPI)

DAPI is a blue, fluorescent immunohistochemical marker that stains the adenine-thymine-rich regions of nuclear DNA in both living and fixed cells [168]. Due to its high-affinity binding with DNA, it is commonly used as a fluorescent counterstain for measuring apoptosis. Normal healthy cells have spherical nuclei with evenly distributed DNA. Morphological changes such as nuclear chromatin condensation, genomic DNA fragmentation, and membrane blebbing occur during the late stages of apoptosis. Chromatin condensation does not occur in necrosis. Thus, chromatin condensation helps to identify apoptotic cells from necrotic cells or healthy cells. This can be observed with DNA-binding dyes such as DAPI. When the dye binds to the compacted chromatin, it is visualized as brighter than the chromatin found in non-apoptotic cells, and the condensed nuclei can be visualized using fluorescent microscopy for qualitative detection and flow cytometry for quantitative analysis. DAPI exhibits a 20-fold enhancement of fluorescence when bound to double-stranded DNA. DAPI can be used along with other color fluorescent dyes such as green or red fluorescence due to its spectral properties. DAPI dye is cell impermeable; therefore, cells must be permeabilized or fixed for the dye to enter the cell and bind to DNA. Another limitation of this marker is that it can enter the live cells only when used at higher concentrations [169].

##### Fluoro-Jade B (FJB)

**Figure 4:**
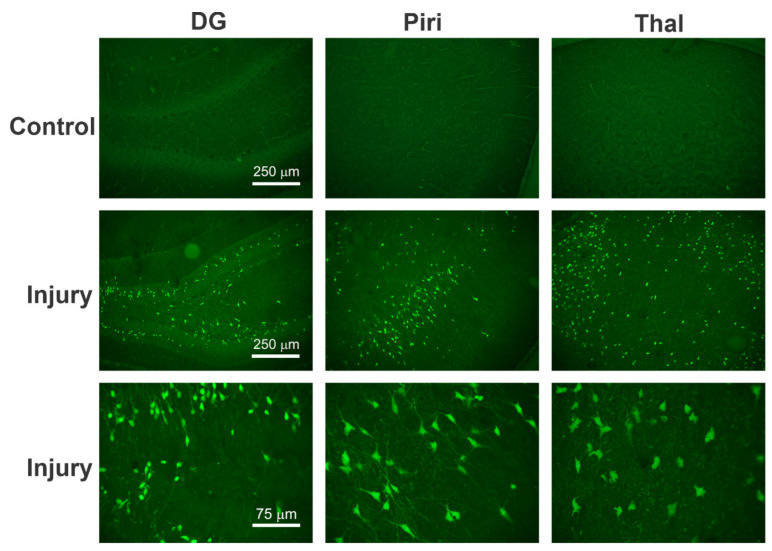
**FJB(+) fluorescent histology of injured and necrotized neurons in the brain**. Representative images of FJB(+) immunostaining depicting dying neurons in the brain sections of rats exposed to the organophosphate convulsant DFP. FJB(+) brain subregions: DG, piriform cortex (Piri), and thalamus (Thal) of control and epileptic rats, 10× (top and middle panel) and 40× (bottom panel) objective. Images were taken via fluorescence-field microscopy. The images were captured based on our lab’s published methodology of DFP-induced neuropathology (Refs. [43,145,146,171]).

FJB is a simple, sensitive, and reliable marker used to localize degenerating neurons and the progression of degeneration. This irreversible neurodegeneration happens due to either an apoptotic or necrotic insult in the brain. FJB is an anionic fluorochrome that can selectively stain dying neurons in the brain [170]. The neurons stained by FJB are detected as individual, bright-green fluorescence, pyramidal-shaped spots lucidly distinguishable from the background under blue-light excitation. Fluoro-Jade is a more sensitive and definitive marker of neurodegeneration compared to conventional procedures such as hematoxylin and eosin or Nissl-type stains [170]. One limitation of using FJB is it only stains the degenerating neurons efficiently with a high resolution and contrast but does not distinguish between apoptosis or necrosis as the mode of cell death [169]. The FJB(+) neurons in different brain regions can be quantitatively analyzed using the optical fractionator cell counting stereology system and qualitatively analyzed using a neuropathology scoring method. The scoring method is carried out by superimposing the traced Nissl-stained sections on the FJB-stained sections. A detailed methodology of FJB histological staining is available in recent papers [43,145,171]. As a representative example, higher-magnification images of FJB(+)-stained healthy and injured rat brain sections are shown (Figure 4).

##### Terminal Deoxynucleotidyl Transferase dUTP Nick End Labeling (TUNEL) 

TUNEL staining is a fast and sensitive method used for in situ identification and quantification of apoptotic cell death or DNA damage. DNA damage or degradation is one of the hallmarks of late phases of apoptotic cells due to the disintegration of nuclear chromatin. This technique relies on the template-independent identification of blunt ends of double-stranded DNA breaks by the terminal deoxynucleotidyl transferase (TdT) enzyme. TdT catalyzes the addition of labeled deoxyuridine triphosphate (dUTP) to the 3′-hydroxyl termini of DNA ends, which is visualized using immunohistochemistry [172]. The nucleus of apoptotic cells treated with the TUNEL mixture exhibits a bright-green fluorescence when observed under a fluorescent microscope [169]. A detailed methodology of the TUNEL assay is available in recent papers [173]. TUNEL staining kinetics are affected by reagent concentration, tissue fixation, proteolysis extent, and accessibility of DNA strand breaks, which differ between tissue types. To avoid false-positive or false-negative results, it is critical to standardize the technique by using tissue sections with DNase treatment as a positive control and tissue sections without TdT treatment as a negative control of apoptosis [174]. DNA damage is not exclusive to apoptosis and can also occur in necrosis. As a result, the accuracy of using the TUNEL assay as a method to specifically detect apoptosis has been called into question in several studies [172,175]. Therefore, it may be necessary to use an additional independent method, in addition to the TUNEL assay, to validate and characterize apoptosis. Immunohistochemical staining for the apoptosis-induced protease caspase-3, Western blots of poly (ADP-ribose) polymerase cleaved by caspases, and the detection of phosphatidylserine on the cell surface with annexin V are examples of such methods [174].

### 2.2. Neuroinflammation

Neurodegenerative disorders are accompanied by a chronic inflammatory response. Neuroinflammation is the defense mechanism used by the host to restore the brain structure and function after a brain injury or neurodegenerative condition. This includes glial cell and complement activation and release of proinflammatory cytokines from glial cells. Neuroinflammation plays a crucial role in the pathophysiology of a variety of neurodegenerative diseases such as PD, AD, HD, and ALS. Astrogliosis and microgliosis have been used as an index for inflammation and neuronal damage. Enhanced expressions of GFAP and IBA1 are commonly utilized to study the activation of astrocytes and microglia, respectively [1,176]. The expression of these markers increases significantly after injury to the CNS.

Microglia are the principal innate immune cells in the brain. Activated microglia play a vital role in neuronal injury. They function as the first responders against pathological attacks. Microglia can transfer to injury sites, remodel synapses, and retain myelin homeostasis. They are heavily involved in the protection against injurious stimuli to the brain by acting as neuroprotective agents expressing pro-inflammatory factors [1]. Microglia activation is crucial for clearing pathogenic factors, supporting tissue repair, and general host defense. Nevertheless, prolonged activation of microglia leads to microgliosis and the overproduction of cytokines and cytotoxic factors, such as reactive oxygen and nitrogen species [177]. Table 5 shows a list of reliable microglial markers used in neuroscience research.

Astrocytes are the most abundant glial cell type in the brain. The extent of the function of astrocytes is diverse. They control blood flow, sustain the blood–brain barrier, provide metabolites as energy for neurons, and modulate synaptic activity. Inflammatory signals released by microglia during neuronal damage can activate pro-inflammatory astrocytes and cause secondary inflammatory responses. However, some pathways activated by astrocyte signaling help to reduce the inflammatory responses acting as a protective function [1]. GFAP and vimentin are the two major intermediate filament (IF) proteins found in the astrocytes [178]. Vimentin is expressed in radial glia and immature astrocytes, and it is replaced by GFAP in differentiated astroglia cells. In response to neuronal injury to the CNS, astrocytes express GFAP, vimentin, and nestin IFs at elevated levels. This overexpression of IFs results in an increased density of IFs in cells [179]. Table 5 shows a list of reliable astrocyte markers used in neuroscience research.

Dendritic branching length, soma size, and total dendritic length can provide extensive information on alterations in neuronal morphometry. Alterations in neuronal morphology of the glial cells can be traced and quantitatively analyzed using advanced scientific software such as Neurolucida and NeuroExplorer [52,180]. The extent of activation of IBA1(+) microglia and GFAP(+) astrocyte elements in different brain regions during neuroinflammation can be quantified by performing area fractionation densitometry using the NIH ImageJ software [43,181]. 

#### 2.2.1. Glial Fibrillary Acidic Protein (GFAP)

**Figure 5:**
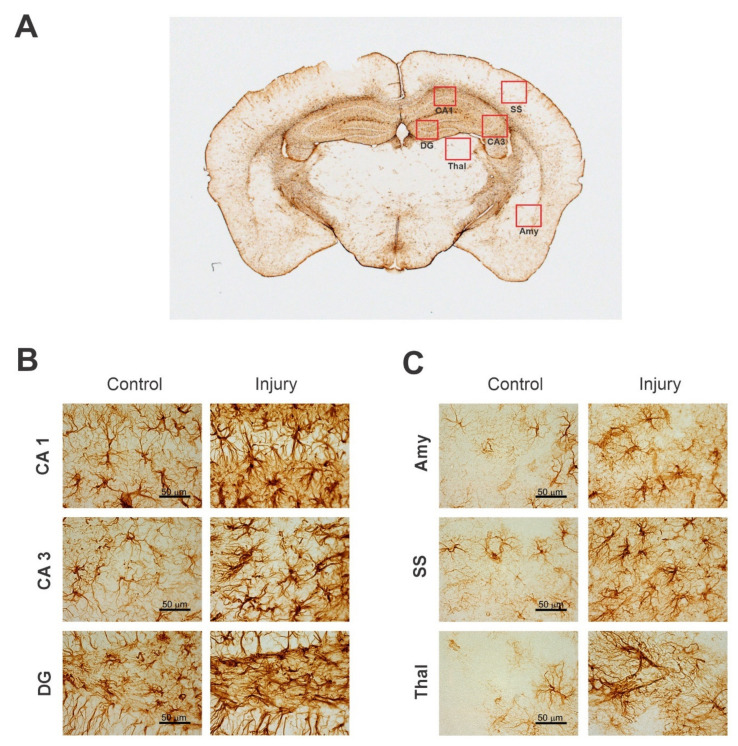
**GFAP(+) astrogliosis in the brain**. Representative images of GFAP(+) immunostaining depicting activated astrocytes in the brain sections of mice after traumatic brain injury. (**A**) Serial slice of GFAP(+)-stained sham mice brain (bregma—1.70 mm), 1.25× objective. The red boxes are regions that are enlarged in panel B and C. (**B**) GFAP(+) contralateral hippocampal subregions: CA1, CA3, and DG of sham and TBI mice, 60× objective. (**C**) GFAP(+) contralateral subregions: amygdala (Amy), somatosensory cortex (SS), and thalamus (Thal) of sham and TBI mice, 60× objective. Images were taken via bright-field microscopy. The images were captured based on our lab’s published methodology of DFP-induced neuropathology (Refs. [43,145,146]).

GFAP is an IF monomeric glial-specific protein rapidly released following brain damage and astrogliosis. The protein size ranges from 40 to 50 kDa [182]. It is mainly present in the CNS, non-myelinating Schwann cells in the peripheral nervous system (PNS), and enteric glial cells. GFAP regulates the cytoskeleton structure and function of glial cells. The expression of GFAP is maintained by nuclear-receptor hormones, growth factors, and lipopolysaccharides. The induction of GFAP protein activation plays a pivotal role in astroglial cell activation (reactive phase) following brain injury and neurodegeneration [183]. After activation, astrocytes rapidly increase the secretion of GFAP. The GFAP protein is released into body fluids, making it a good neuronal biomarker for neurological disorders. It helps to identify patterns of neuroinflammation and subsequent damage [184]. The increased expression of GFAP has been detected in most CNS pathologies, such as TBI, ischemia, and neurodegenerative diseases [157]. A detailed methodology of GFAP histological staining is available in recent papers [43,144]. GFAP is widely identified as an astrocyte differentiation marker. As a representative example, higher-magnification images of GFAP(+)-stained healthy and injured mice brain sections are shown (Figure 5).

#### 2.2.2. Vimentin 

Vimentin is a highly conserved protein (57 kDa) found in tissues of vertebrates, especially in mesenchymal cells [157]. Vimentin belongs to a class III type IF and is considered the only IF type expressed in a range of cells, including astrocytes, fibroblasts, endothelial cells, neutrophils, lymphocytes, and macrophages [178]. Vimentin can form homodimers and heterodimers with other IF proteins such as nestin and destin. The major cellular function of vimentin is the maintenance of cellular integrity, rigidity, and shape of the cell to avoid any cell damage. Vimentin is expressed in radial glial cells during embryogenesis. Vimentin is also involved in the immune response during inflammation [185,186]. Normally, vimentin is non-characteristic for astrocytes in the adult brain, but during neuronal injury, the overexpression of vimentin can be observed in reactive astrocytes in addition to GFAP.

#### 2.2.3. Glutamine Synthetase (GS)

GS is a marker widely used to study the function of the astroglia in clinical and neurobiological studies. GS is a ligase-class enzyme that catalyzes the reaction of ATP-dependent binding of glutamate with ammonia to produce glutamine. It is mainly synthesized in the astrocytes. During the glutamate–glutamine cycle, astrocytes absorb neurotoxic extracellular glutamate, which is converted to non-toxic glutamine via GS. GS is expressed in all the subtypes of astrocytes, making it advantageous over other astroglia markers, such as GFAP. It is also expressed in both large and small cell processes, including the perinuclear cytoplasm, whereas GFAP is only expressed in the cell body and large processes of the astrocytes [157]. Using double-fluorescence GS/GFAP will help to fully assess the astroglia cell population and structural aspects during healthy and pathological conditions. Although GS is mainly expressed in astrocytes, GS expression levels in glutamatergic neurons can rise in neurodegenerative diseases. Thus, it can also be used as a glutamatergic neuronal marker.

#### 2.2.4. S100 Calcium-Binding Protein B (S100B)

S100B is the most extensively studied biomarker to assess patients suffering from TBI and malignant melanoma. S100B is a low-molecular-weight (9 to 13 kDa) Ca^2+^-binding protein highly abundant in the CNS [187]. It is primarily expressed in the cytoplasm and nucleus of astrocytes. However, S100B is also expressed in other glial cell types, such as oligodendrocytes, Schwann cells, ependymal cells, retinal Muller cells, enteric glial cells, and certain neuron subpopulations [188]. S100B plays a key role in astrocytosis, neurite extension, axonal proliferation, and the cytoskeleton structure [115]. When secreted, it has two effects, which are neurotrophic and neuroprotective [189]. However, when S100B is expressed at higher levels, it is shown to be neurotoxic, causing the death of astrocytes. An increase in serum or CSF S100B levels are related to a range of disorders affecting the CNS. Several studies have revealed elevated S100B levels after severe TBI in both serum and CSF [187]. In response to TBI, S100B is released into the bloodstream through the disrupted BBB. This allows for the measurement of S100B levels, which can be used to determine the extent of the TBI [190]. Current evidence suggests that S100B is a promising candidate to be used as a diagnostic and prognostic biomarker for CNS injury. However, the potential therapeutic role of S100B in CNS disorders needs to be elucidated. Further research is required to validate the use of S100B in clinical settings and unrecognized areas of neuroscience.

#### 2.2.5. Ionized Calcium Ion-Binding Adapter Protein-1 (IBA1)

**Figure 6:**
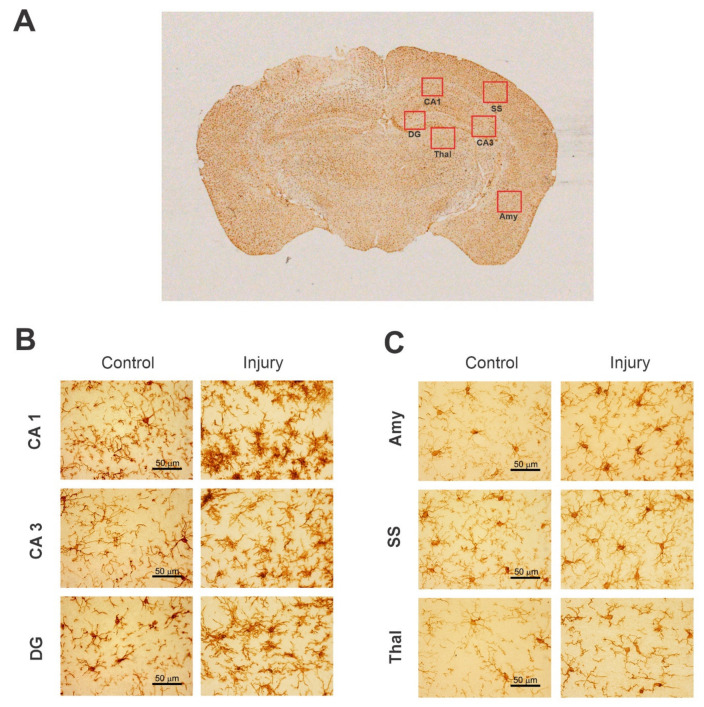
**IBA1(+) microgliosis in the brain**. Representative images of IBA1(+) immunostaining depicting activated microglia in the brain sections of mice after traumatic brain injury. (**A**) Serial slice of IBA1(+)-stained sham mice brain (bregma—1.70 mm), 1.25× objective. The red boxes are regions that are enlarged in panel B and C. (**B**) IBA1(+) contralateral hippocampal subregions: CA1, CA3, and DG of sham and TBI mice, 60× objective. (**C**) IBA1(+) contralateral subregions: amygdala (Amy), somatosensory cortex (SS), and thalamus (Thal) of sham and TBI mice, 60× objective. Images were taken via bright-field microscopy. The images were captured based on our lab’s published methodology of DFP-induced neuropathology (Refs. [43,145,146]).

IBA1, also known as AIF-1 (allograft inflammatory factor-1), is a 17 kDa, actin-binding cytoplasmic protein that is specifically expressed in all microglial subtypes. It is the most common immunohistochemical marker used to determine resting ramified microglia and activated amoeboid microglia, including all intermediate states. The homogenous distribution of IBA1 in the microglial cytoplasm enables the full characterization of the structural features of microglia [157]. IBA1 plays a significant role in regulating the function of microglia. It engages in phagocytosis and microglial cell activation. Membrane ruffling is an important morphological change that takes place when resting microglia turn into activated microglia. Since IBA1 is shown to have actin cross-links in the membrane ruffling of microglia, the activation of microglia is coupled with an increase in IBA1 expression. Due to this activation of microglia, IBA1 staining helps quantify microglia as well as assess the morphological structure during injury phases [176]. A detailed methodology of histological staining is available in recent papers [43,144]. As a representative example, higher-magnification images of IBA1(+)-stained healthy and injured mice brain sections are shown (Figure 6). 

#### 2.2.6. Cluster of Differentiation 4 (CD4)

CNS homeostasis is vital to ensure that the CNS is selectively protected from potentially harmful influences such as microorganisms, toxins, and anomalous immune functions [191]. Bone marrow-derived B-lymphocytes and thymus-derived T-lymphocytes provide faster immune responses by identifying harmful pathogens. CD4+ T-helper cells are a distinct group of classified T-cells that provide immunity to the body. CD4+ cells send signals to other forms of immune cells, such as CD8 killer cells, to destroy the infectious elements. CD4 is a membrane glycoprotein that acts as a co-receptor with the T-cell receptor (TCR) on the T-lymphocytes. CD4 is also found in B-cells, macrophages, monocytes, and dendritic cells. CD4 cells initiate or enhance the early phase of T-cell activation and may serve as an essential mediator of indirect neuronal damage in infectious and immune-mediated diseases of the CNS. During pathogenesis in experimental autoimmune encephalomyelitis, PD, and MS, the transformation of myelin-reactive CD4+ T-cells and their subsequent infiltration across the BBB into the CNS are crucial stages for inflammation [192,193]. CD4+ T-cells can differentiate into Th1, Th17, and Tregs subsets, which give rise to inflammation in the CNS. Cytokines activated by CD4+ cells are responsible for major inflammatory responses [192]. CD4 IHC is helpful for identifying T-cell lymphocytes and related malignant conditions.

#### 2.2.7. Macrophage

The contribution of the innate immune system to the development and advancement of neurodegenerative diseases, such as AD, has been well established. The most common components of the immune system responsible for neurodegeneration are monocytes and macrophages. A specialized population of macrophage-like cells in the CNS has the potential to induce inflammatory responses [194]. Microglia are essential in mitigating or sustaining neuroinflammation. The macrophage colony-stimulating factor (M-CSF) is a hematopoietic growth factor that triggers microglial cells’ involvement in the phagocytosis of amyloid-beta in the brain. This is a precise way of diagnosing AD by measuring the levels of M-CSF. Additionally, the brain perivascular macrophage (PVM) interaction with amyloid-beta induces cerebrovascular oxidative stress during the progression of neurovascular dysfunction [194,195].

#### 2.2.8. Transmembrane Protein 119 (TMEM119)

TMEM119 is an evolutionary conserved, type 1 transmembrane protein specifically expressed by resident microglia under physiological conditions [140]. It was discovered through a comparison of microglial transcriptome datasets. Frequently used microglial markers such as IBA1, CD68, and CD11b fail to discriminate amongst resident microglia and infiltrating macrophages. It is important to discriminate between microglia and macrophages, as microglia arise exclusively from embryonic yolk sac precursors during early embryonic development. This makes microglia ontogenically and transcriptionally distinct from macrophages that infiltrate the human CNS under pathological circumstances. The TMEM119 protein is shown to be expressed only by resident microglia and not by blood-borne macrophages [196,197]. Thus, it is considered a promising selective microglial marker that differentiates resident microglia from blood-derived macrophages in the human and murine brains. Quantitative immunofluorescence and a Western blot analysis are used to measure TMEM119 protein levels. The role of the cell-surface protein TMEM119 in the brain is still unclear [140].

#### 2.2.9. Cluster of Differentiation 45 (CD45)

CD45, also known as protein tyrosine phosphatase receptor type C, is a member of the protein tyrosine phosphatase (PTP) family. PTPs are signaling molecules that regulate a wide range of cellular activities, such as cell proliferation, differentiation, mitosis, and oncogenic transformation. CD45 is a highly conserved cell-surface protein expressed by all hematopoietic cells. CD45 acts as a microglial marker, as it is expressed by both resting and activated microglia [198]. The CD45 biomarker identifies all microglia, regardless of the activation state. Increased expression of CD45+ cells is found in AD pathology [198]. It is also used in many gating strategies to recognize brain immune cell populations using flow cytometry and in single-cell profiling experiments [199].

#### 2.2.10. Cluster of Differentiation 68 (CD68)

CD68 is a 110 kDa transmembrane glycoprotein expressed in microglia. CD68 is a member of the lysosome-associated membrane (LAMP) family and plays a significant role in regulating phagocytosis [200]. CD68 is localized to the lysosomal membrane in microglia and monocytes and is upregulated in actively phagocytic cells. Thus, CD68 is commonly identified as a marker for activated phagocytic microglia [198].

#### 2.2.11. Complement

The complement system is a major component of innate immunity and an appurtenant of adaptive immunity, providing an imperative line of defense against pathogens. It has been involved in the pathophysiology of many debilitating neurological diseases [201]. The complement system comprises around 30 plasma and cell-surface proteins that interact with one another to provoke a series of inflammatory responses involved in the defense against infection [202]. The complement cascade is activated by three initiating pathways (classical, alternative, and lectin). Emerging studies have identified elevated levels of complement components in the CSF, serum, and biopsy specimens of patients with a range of neurological disorders. Thus, complement components at different levels of the complement cascade are used as potential therapeutic targets and predictive biomarkers. The upregulated expression of C1q, the initial protein of the classical pathway, has been identified in many neurodegenerative diseases. IHC in a late-stage AD brain confirmed that the prototypical lesions and amyloid plaques were rich with complement proteins. Accumulation of classical pathway proteins such as C1q, C4, and C3 in the hippocampus and amyloid plaques in the cortex was found in AD postmortem brains [201]. The role of complements in pathophysiology and disease monitoring has also been extensively studied in other neurological diseases, such as myasthenia gravis (MG), MS, AD, PD, HD, ALS, spinal muscular atrophy, stroke, neuromyelitis optica spectrum disorder (NMOSD), and epilepsy. Complement inhibition is an approved therapy for MG and NMOSD [201].

### 2.3. Neurogenesis

Neurogenesis is the term used to describe the birth of new neurons that occur in different brain regions, such as the subgranular zone (SGZ) of the dentate gyrus in the hippocampus or the subventricular zone of lateral ventricles. The main process of neurogenesis is the proliferation and differentiation of neural stem cells into mature neurons, which are integrated into the hippocampal circuitry. The immediate function of these newly formed neurons is not completely known, but they may play a role in encoding temporal aspects of learning and memory [203]. The ability to generate new neurons provides the brain with an important level of plasticity for preserving cellular homeostasis and potentially underlies the response to injury. Studies of adult neurogenesis have revealed extrinsic signals regulating the fate of resident neural stem cells. 

Factors such as physical activity, stress, depression, seizures, and aging can influence neurogenesis. The most common process that causes a decline in the production of neurons is aging, whereas preexisting neuronal populations remain the same while aging. Although the hippocampus is particularly susceptible to age-related alterations and neurodegeneration, finding effective ways to overcome this issue is important in future therapeutic treatments [204].

The status of hippocampal neurogenesis can be analyzed by the stereological quantification of the numbers of newly born neurons (e.g., DCX-positive neurons) in the SGZ-GCL region of the brain. This can be performed using the optical fractionator counting method using NewCast software or the Stereo Investigator system [205].

#### 2.3.1. Doublecortin (DCX)

DCX is a protein that is expressed in migrating neuroblasts and immature neurons. It expedites microtubule proliferation. Newborn neurons express DCX and can maintain the expression for up to 3 weeks. Thus, it is classified as a marker for adult neurogenesis. It can be used to stain the post-mitotic neuronal progenitor cells and early immature neurons. Typically, DCX immunostaining is most intense at neurite extremities and continues into proximal growth cone regions, but not tip regions. A caveat of this marker is that it does not identify all newly born neurons because DCX is not expressed in the newly formed neurons in the neocortex. It is found in newly produced hippocampal, striatal, and olfactory neurons [206]. A detailed methodology of DCX histological staining is available in recent papers [144].

#### 2.3.2. Neuroepithelial Stem Cell Protein (Nestin)

Nestin is a cytoskeletal protein classified as an IF, which is a part of the neural stem cells (NSCs) that develop adult brains. Nestin plays a vital role in pathogenesis. It is expressed in cancers such as osteosarcoma, neuroblastoma, glioma, and melanoma. Nestin can also be classified as a biomarker for invasive phenotypes connected to the subversion in glioblastoma and angiogenesis [56]. Nestin is expressed transitorily in NSCs and disappears during the maturation of neural progenitor cells [206]. In the adult mouse brain, nestin-positive cells can be closely observed in regions rich with proliferation and differentiation of stem/progenitor cells. Nestin is highly expressed during the initial stages of brain development and embryogenesis. The expression of nestin decreases once the cells stop proliferation and differentiation [206].

#### 2.3.3. Neurogenic Differentiation (NeuroD)

The transcription factor NeuroD belongs to a family of basic helix–loop–helix proteins. It is localized in the cytoplasm and the nucleus. NeuroD is expressed in the late stage of neuronal development, and is mostly involved in terminal differentiation, neuronal maturation, and survival. NeuroD is required for the proper differentiation of postnatally born microneurons. Due to its requirement for proper differentiation, NeuroD can be classified as an indicator of neurogenesis, which may serve as a route to understanding the process of neurogenesis. Additionally, it can be used as a marker specific to adult cells in the SGZ and inner granular layer [206].

#### 2.3.4. 5′-Bromo-2′-deoxyuridine (BrdU)

Neurogenesis within the adult CNS is demonstrated using an exogenous cell tracer, BrdU, in combination with endogenous neuronal markers [207]. Specific primary antibodies made against these markers are extensively available and their visualization is achievable with the use of fluorescently tagged secondary antibodies. BrdU is a thymidine analog that incorporates dividing cells during DNA synthesis. Once incorporated into the new DNA, BrdU will pass down to the daughter cells following division. Typically, BrdU is injected intraperitoneally. Different survival times required by the desired experimental timeline will yield data on specific phases of neurogenesis: proliferation, differentiation, and maturation. One limitation of using BrdU is the ambiguous infiltration of the aimed cells with a uniform concentration of the compound. Thus, for experiments requiring measurements of cell proliferation, the Ki67 biomarker can be used as an acceptable alternative [173].

#### 2.3.5. Synaptophysin

Synaptophysin is an integral transmembrane protein with four transmembrane domains and a main, small synaptic vesicle protein, including 7% to 10% of the total synaptic vesicle protein. Synaptophysin acts as a major calcium- and cholesterol-binding protein of synaptic vesicles, having the ability to interact with different synaptic vesicles and motor proteins. Due to its ability to interact with other vesicles, synaptophysin can function in transmitter uptake, vesicular cross-linkage, fusion, and endocytosis [208]. Synaptophysin is a presynaptic membrane protein needed for neurotransmission in hippocampal neurons. Synaptogenesis is an active process in late brain development, but it is the most common protein marker used to detect synaptic plasticity and maturation in the brain [209].

#### 2.3.6. Microtubule-Associated Protein 2 (MAP2)

MAPs are key components of cytoskeleton proteins allied with the microtubule assembly, which is a crucial step in neurogenesis. They bind to microtubules and stabilize them. Examples of MAPs include tau, MAP1, MAP2, MAP3, MAP4, and MAP5. MAP2 is a neuron-specific protein (~280 kDa) that is expressed in neuronally differentiated cells. MAP2 is a family of heat-stable phosphoproteins found in dendrites and the soma of the neurons [210]. Thus, MAP2 is used as a sensitive and specific marker for neuronal damage [211]. MAP2 can only stain the cytoplasm of the neuronal cell body and basal dendrites; it cannot stain the axon, since phosphorylation inhibits the entry of MAP2 into the axon at the axonal hillock [212]. Another limitation of using the MAP2 marker is that it can also be detected in non-neuronal cells such as oligodendrocytes [213]. 

#### 2.3.7. Tau

Tau is a highly soluble, microtubule-associated protein primarily localized in the distal portions of neuronal axons [214]. It is also expressed at low levels in astrocytes and oligodendrocytes [215,216]. Thus, tau can be used as a mature neural marker. Tau is a rod-like structure that forms arm-like projections with microtubules and is firmly bound to the microtubule network. The normal function of tau is to stabilize microtubules and other cytoskeletal proteins in the CNS. Under pathological conditions, confirmational changes and the misfolding of the normal tau protein structure lead to the aberrant aggregation of fibrillary structures in the neurons. Hyperphosphorylated variants of the tau protein fail to interact with microtubules and clump together to form neurofibrillary tau tangles. As a result, the neuronal cytoskeletal network collapses and disrupts the structure and function of the neuron. The soluble tau protein can form insoluble aggregates; this abnormal tau protein aggregation has been demonstrated in a variety of neurodegenerative diseases. Tau mutations can also affect splicing and microtubule-binding efficacy [217]. According to research, axonal outgrowth in the subgranular zone during adult hippocampal neurogenesis requires a dynamic microtubule network, which the tau protein helps to maintain. Thus, tau is a potential biomarker and tool for studying new axons in adult neurogenesis [218]. For additional information about tau, refer to Section 3 in this review.

### 2.4. Mossy Fiber Sprouting (MF)

MF sprouting is the abnormal sprouting of granule cell axons into their dendritic fields, creating new aberrant, recurrent, excitatory circuits. An exact mechanism that shows the processes underlying MF sprouting has not been fully discovered. The sprouting of mossy fibers displays several unusual axonal growth features in the non-plastic adult brain, such as a brain injured by repeated seizures and head trauma [219,220]. MF sprouting is a consequence of repeated seizures and head trauma [220]. It is also considered a hallmark morphological index of temporal lobe epilepsy and bipolar disorder. Sprouted mossy fibers that project from the dentate hilus through the granule cell layer and into the inner molecular layer of the dentate gyrus were first found in patients with temporal lobe epilepsy, and are now known as aberrant mossy fibers [221]. The study of MFS can be helpful to understand the common mechanisms in the pathological reorganization of neuronal circuits. MFS is best visualized by the Timm silver method [222].

#### Timm Staining

**Figure 7:**
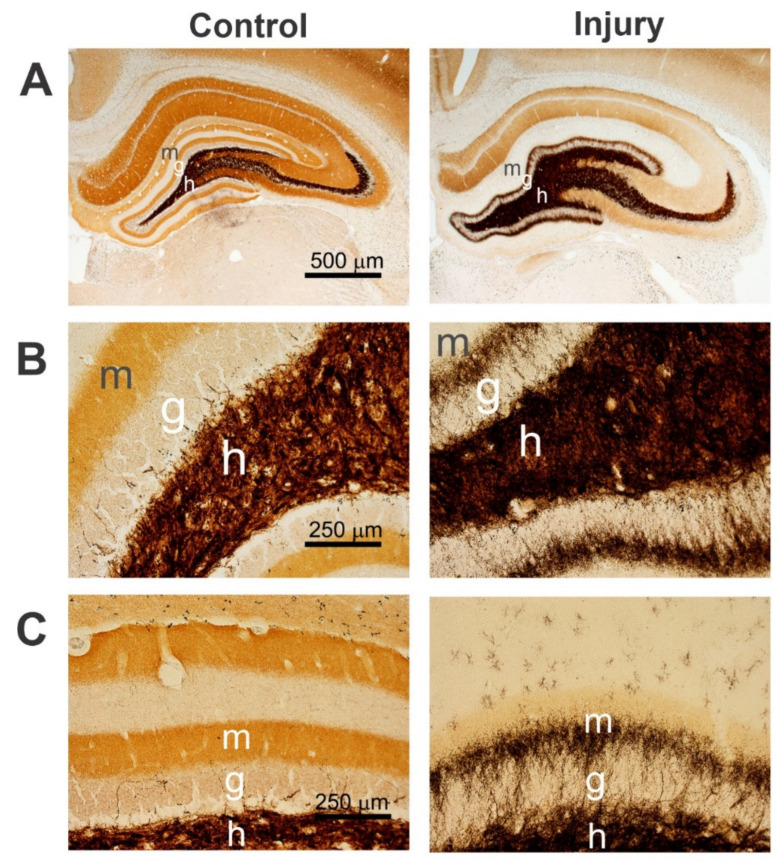
**Timm staining of brain sections.** The extent of aberrant mossy fiber sprouting in DFP-induced epileptic rats is visualized by Timm’s histochemical staining. (**A**) Representative sections of Timm (+) immunostaining of the hippocampus in control and epileptic rats, 1.25× objective; (**B**) and (**C**) magnified views on the dentate gyrus (h = hilus, g = granule cell layer, m = molecular layer) subregion of hippocampus, 20× objective. Images were taken via bright-field microscopy. The images were captured based on our lab’s published methodology of DFP-induced neuropathology (Refs. [43,145,146,171]).

Timm staining is an extremely specific and selective technique for the immunohistochemical detection of zinc and other heavy metals that are present in the CNS. The sprouting of neuronal axons, including mossy fibers that contain zinc, is visualized with Timm staining [173]. Zinc has important roles in the brain, such as controlling neurotransmission and sensory processing, activating pro-death and pro-survival neuronal signaling pathways, and tubulin growth and phosphorylation. Timm staining labels a reserve pool of “labile” zinc that is usually found within synaptic vesicles. The extent of mossy sprouting can be assessed by rating the distribution of supragranular Timm granules in the dorsal and the ventral hippocampus [223]. As a representative example to show aberrant MFS, higher-magnification images of Timm immune-stained brain sections are shown (Figure 7). The extent of MFS in different regions or layers of the hippocampus can be quantified using an area fractionation method of densitometry or a scoring system [224].

### 2.5. Mitochondrial Damage 

Although neurodegenerative diseases are exemplified by the progressive loss and death of neurons, growing evidence in research has highlighted that oxidative stress and impaired mitochondrial function act as formative factors in the pathogenesis of these diseases [222]. Oxidative stress and mitochondrial damage are well known in the pathogenesis of stroke and several age-associated neurodegenerative diseases, including AD, PD, HD, and ALS. Mitochondria, often termed the “powerhouse of the cell”, are double membrane-bound organelles that generate most of the chemical energy in the cell. Apart from energy metabolism, the mitochondria also play a key role in apoptotic and necrotic cell death. Mitochondria possess their own molecular machinery for producing the proteins and enzymes needed for oxidative phosphorylation. Numerous redox reactions catalyzed by enzymes take place in the oxidative phosphorylation process. Any mutation in mitochondrial DNA impairs ATP generation and disrupts the oxidative phosphorylation cascade [225]. Mitochondria are an efficient antioxidant system since they constantly produce free radicals. Mitochondrial damage and disruption of the antioxidant system lead to oxidative stress. 

Oxidative stress is described as the overproduction of reactive oxygen species (ROS), which can provoke mitochondrial DNA mutations, destruct the mitochondrial respiratory chain, change membrane permeability, and impact Ca^2+^ homeostasis and mitochondrial defense systems [226]. ROS include free radicals such as superoxides (O_2_^−^), hydroxyl radicals (·OH), or non-radicals such as hydrogen peroxide (H_2_O_2_). Under physiological conditions, up to 2% of the total cellular mitochondrial oxygen consumed is converted to ROS [225]. The presence of a natural antioxidant defense system actively combats ROS and sustains the normal cellular environment. Cellular levels of ROS are controlled by small-molecule antioxidants (glutathione, taurine, zinc, and vitamin A, E, and C) and antioxidant enzymes (superoxide dismutase, catalase, glutathione peroxidase, etc.). 

Depending on their metabolic requirements, different tissues have different oxygen demands. The brain accounts for more than 20% of total oxygen consumption. The two major types of brain cells, neurons and astrocytes, are mainly responsible for the brain’s substantial consumption of oxygen and glucose [225]. Although oxygen is imperative for the normal function of all living cells, it is potentially toxic in excess. The brain is mainly vulnerable to the effects of ROS due to its high demand for oxygen and its abundance of peroxidation-susceptible substrates [227]. Additionally, the neuronal membranes are rich in polyunsaturated fatty acids, which are highly susceptible to ROS [228]. Therefore, brain cells and neurons require effectual antioxidant protection. The overproduction of ROS in mitochondria or in other sources and changes in the balance of pro-oxidant/antioxidant homeostasis induce oxidative stress and neurodegeneration. Clinical and preclinical findings indicate that neurodegenerative diseases are characterized by decreased levels of antioxidants and antioxidant enzymes in the brain and peripheral tissues. Due to the high reactivity of ROS, they chemically interact with cellular molecules such as DNA, lipids, and proteins. ROS can have a lethal effect on these molecules, causing protein misfolding and aggregation, lipid peroxidation, DNA damage, and mutations, ultimately resulting in necrosis and apoptotic cell death. 

Because of recent discoveries in the connection between mitochondrial dysfunction, oxidative stress, and neurological disorders, novel biomarkers are necessary for the advancement of novel therapeutics.

#### Caspase-3

Caspases are a family of cysteinyl aspartate-specific proteases that are highly conserved in multicellular organisms and function as a key mechanism for apoptosis. Excessive neuronal apoptosis leads to a variety of diseases, such as ALS, AD, HD, PD, and stroke. Among 14 different caspases, caspase-3 is vital during neuronal development and under certain pathological conditions. Caspase-3 is a 32 kDa-sized cytosolic protein that plays a key role in the executive phase of apoptosis. Caspase-3 has been identified as the key facilitator for neuronal programmed cell death and a feature in many chronic neurodegenerative diseases. Apoptosome is a substantial quaternary protein structure formed in the apoptosis process. This apoptosome formation is triggered by the release of cytochrome c from the mitochondria to the cytosol in response to an apoptotic stimulus. Cytochrome c binds to the apoptotic protease-activating factor 1 (Apaf-1) and ATP, which then bind to the inactive pro-caspase-9 to facilitate the apoptosome formation [229]. This can activate pro-caspase-9, which in turn activates and turns the pro-caspase into the effector caspase-3 and initiates a cascade of actions leading to apoptosis. This mechanism leads to cell shrinkage, plasma membrane blebbing, chromatin condensation, and the formation of apoptotic bodies in the CNS. Immunohistochemical analyses of postmortem human AD and ALS brains have revealed increased levels of active caspase-3 activity [230,231]. Caspase-3 expression is an effective marker to study mitochondrial damage leading to apoptosis and the development of novel treatments for a diverse range of neurological disorders [232]. 

### 2.6. Blood–Brain Barrier (BBB) Damage

The BBB is a continuous endothelial membrane, surrounding brain microvessels that preserve cell-to-cell contacts and are enclosed by the mural vascular cells. The cell-to-cell tight junctions of the BBB help to form an impenetrable barrier to peripheral fluids, allowing for the passive diffusion of only water and some lipophilic molecules [233]. The main function of the BBB consists of protecting neurons from the systemic circulation system and regulating the CNS internal milieu, which is needed for proper synaptic and neuron function. The BBB is a selective barrier that protects the brain from endogenous and exogenous chemicals. Many factors can lead to the disruption of the BBB. Any damage to the BBB allows a rapid increase in neurotoxic blood-derived debris, damaged cells, and microbial pathogens that are associated with inflammatory and immune responses. This eventually leads to the activation of multiple pathways for neurodegeneration [234]. BBB damage is found in human patients with TBI, neurodegenerative diseases, and other neurological conditions, and this damage can be replicated in animal models. BBB damage has been depicted in many experimental insults, such as brain ischemia, hypertension, TBI, epilepsy, and irradiation [235]. Biomarkers with different molecular weights can be used as a reliable measurement to detect the damages to the BBB. Under physiological conditions, systemically injected tracers, such as Evans blue (EB), sodium fluorescein (SF), and horseradish peroxidase (HRP) cannot diffuse into the brain parenchyma due to the existence of tight junctions and the restricted transcellular pathway. However, under pathological conditions, the extravasation of these tracers can diffuse into the brain parenchyma [235]. The BBB damage and detection of these markers in brain parenchyma can be experimentally measured via macroscopy, spectrophotometry, spectrophotofluorometry, and electron microscopy. The intravascular infusion of exogenous tracers may provide new insights into both designing approaches for disease management with BBB damage and developing novel trans-BBB drug delivery strategies.

#### 2.6.1. Evans Blue (EB)

The staining most widely used to measure any alterations of the permeability in the BBB is the Evans blue dye (EBD) extravasation. Since EB cannot normally pass through the BBB, it is ideal to use to detect changes in BBB permeability. This dye has a high affinity for serum albumin [236]. EBD can strongly bind to albumin, does not cause metabolic changes, and remains at constant levels the first few hours after the injection of the dye intravenously. The dye then leaks through the damaged BBB and stains the brain lesion where the BBB is disrupted. It is a great marker to identify albumin extravasation and leakage into the parenchyma, and to determine gross microvascular leakage in the brain [237]. An advantage of using the EB tracer is that the region of altered permeability can be accurately detected macroscopically, which is useful in animal studies when lesions with BBB breakdown are studied in specific brain regions. EB has been identified macroscopically as red autofluorescence in brain tissue sections under fluorescence microscopy [235,238]. The extravasated EB can be extracted by different solvents and the concentration is measured by colorimetric assay at the absorbance maximum of 600 to 620 nm [235]. EB can also be measured by spectrophotofluorometry and spectrophotometry in homogenized brain tissue samples. Some of the limitations of using EB as a marker for BBB damage include: (1) a significant amount of free dye may present in the systematic administration of an animal, (2) EB binds to tissues as well as to albumin, and (3) EB has potential lethal toxicity in vivo [235,237]. Nevertheless, EB is an effective marker to study BBB damage as it provides a rapid and reliable assessment of BBB permeability.

#### 2.6.2. Horseradish Peroxidase (HRP)

HRP is also considered to be a widely used technique to visualize the breakdown of the BBB during neuronal disease. It is a useful tracer for IHC. Several types of HRP, such as type II, IV, and VI, are commercially accessible for BBB permeability studies [235]. HRP has both quantitative and qualitative properties. It is a 40 kDa-sized enzyme that catalyzes the oxidation of diaminobenzidine, forming an electron-dense brown reaction product that can easily be detected by visual examination and at the electron microscopic level. The use of HRP in electron microscopic studies gives a better understanding of the structural changes of BBB damage [237]. HRP and its reaction product are restricted by BBB tight junctions in normal conditions. During BBB damage, the BBB becomes permeable to HRP, indicating morphological evidence for a barrier opening. HRP can be measured using a colorimetric assay, and thereby, can be used to quantitate barrier leakage rapidly and simply. The combined use of another marker may provide a more precise and substantial result.

#### 2.6.3. Endogenous Plasma Proteins

Immunohistochemical plasma protein markers, such as albumin, immunoglobulin, and fibrinogen, have been widely used to visualize alterations in the BBB [237]. These proteins can be used to visualize and estimate the permeability of the BBB by monitoring the leakage of these plasma proteins into the brain. Since these proteins are classified as endogenous proteins, they are present inside the cells and they do not need to be injected, avoiding any non-physiological conditions. The cerebrospinal fluid (CSF) and serum quotient of albumin known as Qalb is the most reliable biomarker for measuring BBB permeability. The CSF to serum proportion of immunoglobulin G (IgG), known as QIgG, signifies the IgG penetrability into the CSF from both the blood and intrathecal synthesis of IgG. The measurement of Qalb and QIgG can be used to determine whether IgG or Qalb affects the BBB permeability more [239]. 

#### 2.6.4. Fibrinogen

Fibrinogen is a 340 kDa-sized blood coagulation plasma glycoprotein deposited in the brain during neurological diseases and traumatic injuries in connection with BBB disruption, including MS, TBI, AD, PD, HD, ALS, and stroke [240]. Fibrinogen is synthesized by hepatocytes in the liver and secreted into the blood circulation as a soluble homodimer. Fibrinogen primarily maintains hemostasis by platelet aggregation, leading to blood clot formation. Because of its critical role in hemostasis, changes in fibrinogen levels can have serious pathological consequences. Fibrinogen is linked with neuroinflammation, neuronal damage, and the entry of immune cells into the nervous system. Fibrinogen can stimulate CNS inflammation, inducing scar formation, which results in the decline of cognitive ability and inhibition of repair. Fibrinogen is not expressed in the healthy brain due to the presence of an intact BBB. Following BBB disruption, fibrinogen can enter the parenchyma of the nervous system, where it is transformed into insoluble fibrin by the perivascular tissue factor and procoagulant proteins that are abundant after injury [240]. Hence, it has been widely employed as a reliable marker to study BBB disruption in human tissue and animal models. Immunohistochemical and electron microscopy detection of fibrinogen/fibrin deposition in the CNS has been widely used as a measurement of BBB dysfunction in several studies evaluating neurological diseases linked with BBB disruption [240,241]. Since fibrinogen has many binding sites for cellular receptors and proteins expressed in the nervous system, it can also serve as an imaging and fluid biomarker, as well as a therapeutic target for neurological diseases [242]. Fibrinogen leakage caused by BBB disruption is a common mediator of neurodegeneration and CNS innate immunity activation, making it a promising therapeutic marker for neurological diseases associated with BBB leakage.

#### 2.6.5. Sodium Fluorescein (SF)

SF is a small-molecular-weight tracer widely used for the assessment of BBB permeability. SF was the first visualizable small-molecular (376 Da) marker to be introduced into the BBB [237]. It is a fluorescent, synthetic, organic compound that, when combined with sodium salt, becomes a water-soluble dye with major blue and green excitation peaks. The blue fluorescence excites in the range of 460–500 nm, and the green emission peak is in the 540–690 nm range. The fluorescence is useful because it accumulates in the cerebral regions during BBB disruption. Hence, images can be visualized based on excitation patterns. SF is also useful in improving brain tumor visualization [243]. Advantages of using SF as a BBB permeability marker are that it is nontoxic, inexpensive, small, easily diffusible, and visible at extremely low concentrations [235]. SF binds to proteins not as strongly as EB and appears to be an effective low-molecular-weight marker for BBB studies [237]. SF can pass through BBB much more readily due to its lower molecular weight than other large-molecular-weight tracers. Another advantage of using SF is that it is less toxic than Evans blue or HRP. A limitation of using SF to study BBB disruption is that it cannot be accurately detected via visual examination [235]. It is recommended that alterations in BBB permeability to SF may be the earliest and the most subtle indicator of BBB disruption. Thus, SF can be used as the tracer of choice or expedient complement to prevailing tracers.

#### 2.6.6. Dextrans 

Dextrans are formed by varying chain lengths of glucose molecules, ranging from 4–2000 kDa. These compounds are highly stable and sensitive; thus, they are reliable markers for establishing the level of damage to the BBB. Since these molecules are not endogenous, they can be introduced to the life cycle of any disease at any given stage to determine the time of BBB disruption without causing any negative effects. They are usually readily available and labeled with fluorophore or biotin. The smallest biotin marker for dextran is ethylenediamine (286 kDa), which is commonly used for quantitative studies of BBB permeability. The advantage of biotin labeling is that it can be visualized using both light and electron microscopical levels [211]. They can also be quantified in CSF.

## 3. Blood Biomarkers of Neuronal Injury and Neurodegeneration

Neurodegenerative diseases, such as AD and PD, require a lengthy prodromic period, making it difficult to identify early pathological changes as they are asymptomatic. Therefore, biomarkers rather than clinical indications are required for early diagnosis and longitudinal follow-up of future cases. The excessive cost of neuroimaging biomarkers and the invasiveness of lumbar punctures for CSF makes it unsuitable for routine applications in clinical settings. Thus, there is a strong need to develop dependable, less invasive, and more accessible diagnostic methodologies, such as blood-based biomarkers, that can be used for screening and to raise the efficacy of clinical trials. In humans, nearly 500 mL of CSF is absorbed daily into the blood, making blood a suitable source of neurodegenerative disease biomarkers [244]. Easily accessible blood tests provide valuable information about the pathophysiological basis of neurological diseases. Plasma is a complex body fluid comprising proteins, lipids, peptides, and metabolites that reveal the physiological and pathological activity in several body organs, including the CNS. Changes in plasma proteins, plasma metabolites, or even the microRNA levels can reflect mechanisms of AD pathology, including neuronal cell death, inflammation, and oxidative mechanisms, or can provide valuable insights into the novel pathways of the disease etiology. Numerous potential plasma biomarkers, such as the tau, neurofilament light chain (NfL), and brain lipid-binding protein (BLBP), for neurodegenerative illnesses have been found; however, applications of these candidate biomarkers for widespread use in clinical settings have not yet been established. Some blood biomarkers of neurological diseases remain experimental, with few validated clinical applications. In therapeutic trials, neurological blood markers, such as p-tau 181 or Aβ42 for AD and NfL for ALS, are used in routine clinical diagnosis [245,246,247,248,249]. Several blood-based biomarkers have advanced into clinical practice. The FDA recently approved the detection of the glial fibrillary acidic protein (GFAP), an indicator of glial injury, and ubiquitin carboxy-terminal hydrolase L1 (UCH-L1), an indicator of neuronal injury, for assessment after suspected injury. Additional molecules indicative of cellular damage or inflammation continue to actively be investigated. Future research will help with translating the effective incorporation of plasma biomarkers into clinical practice, including validation across diverse patient populations and the incorporation of approved biomarkers into clinical guidance in different care settings.

### 3.1. Tau

Tau is a protein that is highly expressed in the CNS, where its key role is stabilizing microtubules, which are key elements of axonal transport and signal transduction. Differentiation in tau expression levels is seen in many neurodegenerative disorders. Tau is affected in a variety of ways, such as in mutations at the genetic level, tau isoforms caused by changes in mRNA-level alternative splicing, and increased levels of total and phosphorylated tau proteins. All these alterations lead to an increased risk of neuronal injury and neurodegenerative disorder, commonly named tauopathies. The molecular diversity of tau allows it to be detected in many diverse backgrounds, making it a reliable biomarker for diagnostics. Quantifying tau in CSF using techniques such as mass spectroscopy has become an important early detector for AD [250].

Tau is differentiated into phosphorylated tau (p-tau) and total tau (t-tau) proteins. p-Tau is associated with the formation of neurofibrillary tangles in the brain, whereas t-tau is an indication of the severity of neurodegeneration in AD patients. Previous studies have shown that t-tau in the CSF is three times higher in AD patients than in normal aging individuals. Moreover, levels of CSF t-tau can be used to measure acute disorders such as stroke, brain disorder, and chronic neurodegenerative diseases [250].

### 3.2. Neurofilament Light Chain (NfL)

The principal function of neurofilaments is to maintain the axon structure and transport. Neurofilaments are exclusively expressed in neurons in three types of isoforms: NfH, a high-molecular-weight subunit (180 to 200 kDa); NfM, a middle-molecular-weight subunit (130 to 170 kDa); and NfL, a low-molecular-weight subunit (60 to 70 kDa) [251]. NfL is a neuronal cytoplasmic protein that is vastly expressed in large-caliber myelinated axons. NfL can be reliably quantified in blood using ultrasensitive single-molecule array (Simoa) technology. Increased levels of NfL in CSF and blood are consistent with the degree of axonal damage in a variety of neurological disorders, including MS, ALS, frontotemporal dementia, and familial and sporadic AD. Particularly, for ALS, NfL can be used as a successful biomarker to show how the transition from pre-symptomatic to symptomatic stages occurs as the disease progresses [252]. However, NfL levels increase in normal aging and many other CNS disorders including Down’s syndrome with no clinical evidence of AD. Therefore, it is a nonspecific marker for neurodegeneration [38].

### 3.3. Brain Lipid-Binding Protein (BLBP)

Lipids are found abundantly in the brain because they are essential for the expansion of neurons and the construction of myelin. Proper lipid control in the brain is needed to maintain proper functioning. Lipid homeostasis changes are known to cause or act as risk factors for neurodegenerative diseases [253]. Lipid-binding proteins play a key role in the maintenance of brain function through aging. The brain lipid-binding protein (BLBP) belongs to a family of small, highly conserved, cytoplasmic proteins named fatty acid-binding proteins (FABPs). BLBP plays a significant role in fatty acid uptake, transport, and metabolism. BLBP proteins are present in the nucleus and cytoplasm [254]. BLBP has potential morphogenetic activity in the nervous system, which can be used as an identifier in neuroblastic tumors. In addition, a BLBP named apolipoprotein E is known to have a significant correlation with the progression of AD [253,255]. BLBP is expressed in radial glia in both the postnatal cerebellum and embryonic ventricular zone [254]. Therefore, BLBP is used as a serum protein biomarker for radial glia/astrocytes in the developing brain [50]. 

### 3.4. MicroRNAs (miRNAs)

miRNAs are a class of small, endogenous, and highly conserved non-coding RNAs that are an average of 17–25 nucleotides in length [10,256]. miRNAs play vital roles in protein synthesis and regulating gene expression in the post-transcriptional phase [257]. miRNAs regulate more than one-third of the human gene expression. The main function of miRNAs is to adjust protein levels and regulate mRNA degradation. The literature reveals that approximately 70% of all miRNAs are expressed in the cerebellum, midbrain, hippocampus, and frontal cortex [258,259]. miRNA plays a critical role in brain morphogenesis, synapse formation, neural differentiation, neural maturation, and neurogenesis [259]. There are a few miRNAs that are only expressed in a brain-specific or brain-enriched manner [258]. These endogenous molecular regulators appeared to be distorted in different pathologies, including TBI [10]. Thus, they have been widely reported as new potential biomarkers for the diagnosis and prognosis of a range of diseases.

## 4. Imaging Biomarkers of Neuronal Injury and Neurodegeneration

Neurodegenerative diseases are profoundly diagnosed pathologically. Hence, an autopsy is required for a conclusive diagnosis of a neurodegenerative disease. There is a rising need for dependable in vivo measurements to advance the fundamentals of characterizing neurodegenerative disorders. Identifying novel therapeutics will help better visualize the disease progression and underlying pathology of neurodegeneration and injury. Recent advancements in various molecular brain imaging techniques have allowed for the identification of neuropathological changes at initial stages in neurological disorders and allowed us to longitudinally monitor the progression without an autopsy [260]. Neuroimaging has become a gold standard in the clinical evaluation of individuals suspected of having a neurodegenerative disease. Neuroimaging biomarkers are intriguing not only because they are noninvasive, but also because they are routinely performed as part of a patient’s routine medical care [261]. Newly developed radioactive isotopes and contrasting agents increase the efficiency of MRI, MRS, and PET scans. Nevertheless, the cost factor and injection of radioactive material restrict biological applications. As technology advances, new diagnostic approaches will be made to further improve the capabilities of imaging biomarkers [262].

### 4.1. Computed Tomography (CT)

A neurological CT scan uses ionizing radiation (X-rays) to generate 2D images. It is a noninvasive imaging technique that is commonly used to examine structures in the brain, organs, bones, tissues, and spinal cord. The series of 2D images taken during a CT scan can be stacked together to create a 3D image, allowing for a much more comprehensive view than traditional X-rays can offer [263]. A CT scan can assist in proper diagnosis by revealing areas of the brain that are affected by neurodegenerative diseases. CT scans can be used to quickly detect a hemorrhage in the brain or to detect different vascular irregularities, brain tumors and cysts, brain damage causing epilepsy, and much more [264]. A contrast dye can be injected into the bloodstream to focus the distinct tissues in the brain that are affected during disease progression. There is a slight risk of cancer by exposing the patient to radiation used in CT scans. CT scans can also be cost-prohibitive to groups of patients. CT scans do not require any recovery time after the procedure. It is an especially useful imaging biomarker to find an underlying brain pathology [263].

### 4.2. Magnetic Resonance Imaging (MRI)

MRI is a widely used, noninvasive imaging technique that produces three-dimensional anatomical images of the brain and other cranial structures [15,260]. MRI uses robust magnetic fields and radio waves to generate detailed images of any structure from any angle. It is becoming an increasingly popular in vivo imaging procedure to further investigate anatomic, functional, and neuronal lesions in the brain [15,261,263,265]. A special type of MRI known as functional MRI (fMRI) can be used to generate images of blood flow to certain areas of the brain and to examine neurodegeneration from head injuries or other conditions [261]. Additionally, structural MRI (sMRI) provides static anatomical information about the brain. With the development of MRI scanning, it is easier to observe volumetric changes in disease progression in several damaged zones of the brain such as the hippocampus, amygdala, entorhinal cortex, and so on [15]. Both fMRI and sMRI combined help in excluding brain lesions, determining patterns of atrophy, and assessing vascular burdens during neurodegeneration. MRI is a useful technique for identifying an early diagnosis and it presents minimal risks to the patient, because unlike CT scans, MRI produces images without using ionizing radiation. It can also give clear, detailed images of soft-tissue structures that cannot be achieved through other imaging techniques. Potential risks from an MRI include an allergic reaction to the contrasting material or feelings of claustrophobia. Since MRI uses strong magnets, it is not safe to be used in patients who have metal or electronic devices in the body. The metal present in the body can be attracted to the magnetic field or alter the MRI image. The impact from MRI on a fetus is not yet understood. Therefore, MRI is not safe during pregnancy. MRI can also be cost-prohibitive to groups of patients. However, the images produced by the MRI scan are beneficial for efficient measurements in detecting neurodegeneration [262].

### 4.3. Positron Emission Tomography (PET)

A PET scan provides 2D and 3D pictures of the brain by measuring radioactive isotopes that are injected into the bloodstream. The most used radionuclide is fluoro-deoxy-glucose (FDG), which measures metabolic activity in the brain. Additionally, PET scans can also be used to detect tumors, show blood flow, and evaluate seizure disorders that cannot be seen using other medical therapies. PET is especially capable of measuring lesions that are not visible on MRI scans [266]. PET is capable of measuring neuroinflammation and can distinguish components of the neuroimmune response [261]. During a PET scan, a low-level radioactive isotope called a “tracer” is injected and the tracer’s uptake in the brain is measured. Using different compounds, more than one brain function can be measured and traced. The PET scan is painless and uses lesser amounts of radioactivity. The use of PET in a clinical setting is limited due to factors such as machinery costs and radiochemical laboratories. Once these limitations are bypassed, PET is an essential technique in the diagnosis and development of therapies and the prediction outcomes in various neurological diseases such as PD, HD, MS, and dementias [267].

### 4.4. Magnetic Resonance Spectroscopy (MRS)

MRS imaging is exceedingly adaptable in evaluating many different parameters of anatomic, physiologic, and metabolic activity in the brain. It provides a means of measuring different changes in cerebral metabolites relevant to neurodegeneration in vivo. Most clinical MRS research focuses on the metabolites that coincide with different neurodegenerative diseases. Brain metabolites can function as surrogate indicators for different symptomatic processes such as neuronal damage, glial proliferation, inflammatory changes, and loss of membrane integrity [268]. MRS-derived markers have an extremely specific activity that makes it easier to distinguish between normal and abnormal changes in the brain. MRS has the potential to detect early metabolic changes in AD and ALS, whereas MRI does not. MRS is also readily accessible, inexpensive, and gives no exposure to radiation. These factors of MRS make it a vital imaging biomarker to determine pathological and clinical changes in neurodegenerative diseases [269].

### 4.5. Fluid-Attenuated Inversion Recovery (FLAIR)

FLAIR is regarded as one of the most useful contrast techniques that is used to investigate white matter (WM) diseases such as MS, infarctions, subarachnoid hemorrhages, and other head injuries. FLAIR is an MRI sequence that generates strong T2 weighting and reduces the contrast between gray and white matter. It is used to recognize various pathologies near the CSF space due to its capability to suppress the CSF signal, making the region clearer [270]. FLAIR images produce increased lesion-to-background CSF contrast and improve the visibility of lesions. Since FLAIR has an extremely high sensitivity to white matter deformities, brain imaging with T2 weighted FLAIR can be used to measure changes in the lesion load, including in the cortical and periventricular regions which are typically difficult to see on traditional imaging techniques. However, the information gained on the pathology of brain lesions is not specific. 2D-FLAIR is most used in clinical studies [271].

## 5. Conclusions and Future Perspectives

Neurodegenerative diseases and neuronal injury create serious health and socioeconomic problems in the world and cause high morbidity and mortality. Neurodegenerative diseases consist of a spectrum of afflictions characterized by progressive neuronal, synaptic, and axonal loss. Neurodegeneration with the progressive loss of major neurons is a core feature of many brain diseases, including not only classical degeneration conditions, such as AD, PD, HD, and ALS, but also neuroinflammatory diseases such as MS and neuromyelitis optica. In many neurological conditions, an initial or persistent inflammatory feature is succeeded by neurodegeneration. The consistent visualization and labeling of key markers and pathways are inevitable methods for assessing the progression or refractoriness to interventions. Despite decades of research, AD and other dementias have limited treatment options. Moreover, most therapies are used to treat symptoms rather than the underlying disease. There are few “disease-modifying” therapies for chronic brain disorders. 

Research over the past 20 years has provided a better understanding of molecular and cellular aspects of brain disorders, mostly from experimental models and clinical verifications. Genomics, proteomics, and RNAseq research has provided numerous early markers and biomarker studies in the brain, CSF, and plasma, such as markers of inflammation, immune dysregulation, apoptosis, and cell death pathways in neuropathogenesis. A final pillar of evidence for disease or its progression is provided by biomarker monitoring. However, the consistent visualization of neurons and glia in the brain is crucial for the quantitative analysis of the cellular pathophysiology changes that occur in neurological diseases. Biomarkers are useful to examine the severity of these diseases and monitor the diagnosis and prognosis of different pathologies. Biomarker research in neurodegenerative disorders is an intriguing and fast-developing area. As outlined in this article, there is a great need for reliable experimental and clinical biomarkers of neurodegeneration and neuronal injury for monitoring therapeutic interventions. 

Despite the development of highly predictive markers for studying brain disorders, limited progress has been made in addressing the validation and optimization of disease-specific biomarkers. Neuronal loss and abnormalities in glia and oligodendrocytes are primarily evident as part of disease progression. Neuroinflammation is central to many forms of both acute and chronic neurological conditions. Therefore, there are many potential therapies in development for halting or attenuating such chronic inflammation and neuroimmune systems; these treatments, however, have not been extraordinarily successful in preventing progressive brain conditions, such as AD, PD, and MS. Further investigations of signaling cascades of neuronal loss and inflammation driven by CNS-resident cells—including astrocytes and microglia—are, therefore, warranted to identify novel potential biomarker opportunities. 

There are few validated biomarkers for accurately predicting neurodegeneration in people at risk for such conditions. Many biomarkers discussed have some limitations; thus, it is unlikely that any single biomarker can accurately measure the progression of a disease. A combination of multimodal biomarkers will facilitate the full evaluation of neurological conditions. Although neuroscience research is rapidly improving, our understanding of the pathophysiology of neurological diseases and the exact mechanisms and factors underlying them remains unclear. There are accelerated efforts to identify and validate a toolkit of biomarkers for the precise and objective diagnosis and categorization of a brain injury or other neurodegeneration conditions. Launching biomarkers to monitor the treatment after injury and refine future interventions is an active area of investigation and clinical implementation. 

The current landscape classifies four major types of biomarkers, including blood-based, immunohistochemical-based, imaging-based, and neurophysiological markers. Neuroimaging is a power tool to characterize the nature, location, and extent of injury or changes after a suspected brain disease. A variety of blood-based biomarkers have also been identified, with a few now reaching clinical practice. For example, the FDA recently approved the detection of GFAP (an indicator of glial injury), and ubiquitin carboxy-terminal hydrolase L1 (UCH-L1, an indicator of neuronal injury) for assessment after brain injury. In addition, electrophysiological measures such as EEG, which measures electrical activity in the brain, and wearable biosensors are being evaluated for their utility in assessing neurological conditions. A single biomarker is unlikely to capture the full pathophysiology of any brain disease, particularly the dynamic and long-term neurological conditions. Approaches that include multiple biomarkers are likely to be needed. Continued efforts to identify and validate new markers will also be needed for translation and effective incorporation of biomarkers into clinical practice in different care settings.

Breakthrough advances in biology, such as microarray, next-generation sequencing, 3D cell culture models of the human brain, and accelerating neuronal biomarker discovery have paved the way for the incorporation of precision medicine in clinical neurology. Precision medicine integrates basic research and clinical practice to build a platform that can better direct tailored patient care based on the unique genetic makeup, biomarker characteristics, and bioinformatics which underpin the personalization of medical care. The application of precision medicine to the treatment and prevention of neurological disorders appears to be extremely promising in contrast to the traditional drug treatment approach.

There are many questions that need to be answered by further research investigations on biomarkers, including: (a) What information can biomarkers provide the neurology community and how can a toolkit of biomarkers inform the research protocols? (b) What needs are not yet met with the current biomarkers in regard to neurological conditions? (c) What are the contexts of use of multiple markers where biomarkers will impact research and interventions? (d) What can the neurology community learn from the cancer community’s experiences in clinical validation and use of biomarkers? (e) How can biomarkers be incorporated into a new diagnostic and therapeutic scheme for neurodegenerative conditions? (f) What approaches are needed to validate and incorporate novel biomarkers into an effective therapeutic approach? (g) Where are we now in terms of utilizing biomarkers and what have been the outcomes and lessons learned for precision diagnostics? Further discoveries in neuronal biomarkers will allow for better characterization and open new insights into personalized therapeutic approaches for the prevention of chronic neurological diseases.

## Figures and Tables

**Table 1:** An overview of human brain regions, cell types, and their functions.

*Brain Region*	*Function*	*Cell Types*	*Function*
		***Neurons***	The two main subclasses of neurons are projection (principal) neurons and interneurons.
Olfactory bulb	Receives input from the olfactory neurons and projects to the olfactory nucleus, piriform cortex, and amygdala.	Principal neurons	Main signaling units in the brain, communicating with each other via synapses.
Cerebral cortex	Consists of excitatory projection neurons and inhibitory interneurons. It processes and filters sensory information and sends information to motor neurons in the spinal cord.	Interneuron	Provide local interconnections between projection neurons to control communication.
Hippocampal formation	Associated with learning and memory. The main cell types are pyramidal projection neurons, granule cells, and interneurons.	***Non-neurons***	Support and promote the proper function of neurons. These include endothelial cells lining blood vessels, ependymal cells lining the ventricular walls, and glial cells. Glial cells include oligodendrocytes, microglia, and astrocytes.
Amygdala	Located deep within the temporal lobe and is associated with emotions, such as fear, and with emotional learning.	Oligodendrocytes	Insulate neuronal axons for faster signal conduction.
Basal ganglia	A collection of subcortical nuclei, such as the striatum, globus pallidus, and substantia nigra, which are involved in movement control, learning, addiction, and reward.	Microglia	Act as brain macrophages (with a hematopoietic origin) for protecting the brain from infection and injuries.
Hypothalamus	Integrates the two-way communication between the brain and the rest of the body. It regulates the secretion of pituitary hormones, food intake, temperature, and circadian rhythms, and senses blood-borne hormones.	Astrocytes	Involved in numerous functions, such as maintaining the BBB, homeostasis, neuronal growth, and neurotransmitter recycling.
Thalamus	Processes sensory and motor information destined for the cortex and plays a critical role in sleep and consciousness.		
Midbrain	Participates in the processing of auditory and visual information and the regulation of motor behavior.		
Pons	Involved in breathing, eye movement, and various other senses.		
Medulla oblongata	Contains several motor nuclei that control autonomic functions, including respiration, vomiting, sneezing, heart rate, and blood pressure. It also incorporates sensory nuclei that receive input from the vagus nerve.		
Cerebellum	Contains large Purkinje cells, is associated with motor control, motor learning, and coordination, and is important for certain cognitive functions.		

**Table 2:** Potential biomarkers in acute neuronal injuries and neurological disorders.

Disease	Pathological Features	Biomarkers	References
*Acute neuronal injuries*:			
Acute ischemic stroke	Blockage of cerebral blood vessels, leading to neuronal necrosis, cell death, and inflammation	S100B, GFAP, BNP, MCP-1, caspase-3, NSE, MMP-9, NMDA-R, PARK7, CRP, IL-6, TNF-α, procalcitonin, MMP-8, GABA, UCH-L1, sNfl, miRNA, NfL, CT, MRI, MRS	[7,8,9]
Traumatic brain injury	Secretion of inflammatory cytokines by activated glial cells leading to neurodegeneration and neuronal dysfunction	NeuN, PV, GFAP, IBA1, Timm, DCX, FJB, S100B, UCH-L1, MAPT, NSE, AMPc, MBP, tau, IL-1B, IL-6, IL-8, TNF-α, IFN-γ, PNF-H, NMDAR, all-spectrin, Hsp70, AQP4, SBP, miRNA, MRI, CT	[10,11]
Encephalitis	A viral/bacterial infection or immune system malfunction leading to brain inflammation	YKL-40, IL-6, IL-8, TNF-α, Aβ38, Aβ40, β2M, Aβ42, GFAP, sTREM-2, NfL, t-tau, p-tau, MRI, CT	[12,13]
Hemorrhagic stroke	Rupturing/bleeding of brain blood vessels leading to neuronal necrosis, cell death, and inflammation	S100B, GFAP, NSE, MMP-9, MRI, CT	[9,14]
***Chronic neurological conditions***			
Epilepsy	Absence or excess signaling of neurons results in unpredictable, spontaneous, and recurrent seizures leading to neurodegeneration, BBB damage, and inflammation	miRNA, NeuN, PV, FJB, GFAP, IBA1, Timm, DCX, IL-1, IL-6, TNF-α, UCH-L1, NSE, MMP-9, S100B, MRI, CT, SPECT	[15,16]
Neuropathic pain	Lesion or disease affecting the somatosensory nervous system leading to altered and disordered sensory signal transmission	IL-1β, IL-6, IL-2, IL-33, CCL3, CXCL1, CCR5, and TNF-α, sICAM-1, CRP, miRNA, TSPO, PET, MRI	[17,18,19]
Migraine	Activation of the trigeminovascular system	TNF-α, homocysteine, somatostatin	[20]
Parkinson’s disease	Intracellular aggregates of α-synuclein in the form of Lewy bodies and Lewy neurites leading to the loss of dopaminergic nigrostriatal neurons in the substantia nigra pars compacta	α-Synuclein, miRNA, orexin, caspase-3, TCS, NfL, Aβ42, p-tau, CRP, D3R, 8-OHG, YKL-40, MCP-1, MHPG, GCase, GlcCer, cathepsin D, miRNA, DJ-1, PET, MRI, SPECT, TCS, DWI	[21,22]
Frontotemporal dementia	Accumulation of different forms of aberrant tau aggregates in the brain leading to the atrophy of the frontal lobe	Aβ42, t-tau, pT181-tau, pS396-tau, NfL	[23]
***Neurodegenerative diseases***			
Multiple sclerosis	Inflammatory lesions create multiple plaques in the gray and white matter of the brain and spinal cord, leading to neuronal demyelination, axonal degeneration, and neurological dysfunctions	Tau, NFL, NFH, CXCL13, miRNA, ApoE, MBP, OPN, NCAM1, NGF, CNTF, GFAP, tau, S100B, Ferritin, CD163, YKL-40, Kir4, MRI	[24,25,26,27]
Huntington’s disease	Expansion of CAG repeats in the huntingtin gene leading to progressive degeneration and atrophy of the striat um; loss of striatal neurons and cell death	mHTT, tau, NFL, NFH, miRNA, TDP-43, NPY, PDE10A, MRI, PET	[28,29,30]
Alzheimer’s disease	Extracellular aggregates of amyloid β (Aβ) plaques and intracellular neurofibrillary tangles (NFTs) made of hyperphosphorylated tau protein and inflammation; leading to synapse dysfunction, neuronal cell loss, and brain atrophy	Tau, p-tau, NfL, FABP, Aβ1-42, MCP-1, YKL-40, TREM2, neurogranin, MRI, PET, FDG-PET, amyloid PET, NSE, VLP-1, HFABP, albumin, GFAP, α-synuclein, t-tau, pT181-tau, pS396-tau	[23,31]
Amyotrophic lateral sclerosis	Progressive loss of motor neurons in the motor cortex, lower cranial brainstem motor nuclei, and anterior horn cells of the spinal cord leading to voluntary muscle and cognition impairments	NfL, phospho-NfH, TDP-43, tau, Aβ, p-tau, GDNF, TSPO, CHIT1, CHI3L2, f4-HNE, ferritin, MMP-2, MMP-9, IL-6, IL-8, PGE2, MCP-1, fMRI, PET, DTI, SPECT	[32,33,34]
Creutzfeldt–Jakob disease	Accumulation of misfolded prion proteins (PrPs), and spongiform changes in the brain leading to neurodegeneration and cell death	MRI, DWI, FLAIR, PET, NSE, tau, NfL, PrP	[35]
Multiple system atrophy	Abnormal accumulation of misfolded hyperphosphorylated α-synuclein in the brain, leading to the progressive loss of oligodendroglia and neuronal death	α-Synuclein, NfL, tau, miRNA, MRI, DTI, PET	[36,37]
Down’s syndrome	Overexpression of the APP gene leading to accumulation of brain Aβ and tau pathologies typical of AD	Tau, p-tau, NfL, GFAP, PET, MRI, Aβ42/40	[38]
Spinal muscular atrophy	Progressive loss of motor neurons in the spinal cord and motor nuclei in the lower brain stem resulting in muscle weakness	SMN, NfL, creatinine, GFAP, MRI, miRNA	[39,40]

**Table 3:** List of potential biomarkers of neuronal injury and neurodegeneration.

*Biomarker*	*Target*	*Functional Role*
NeuN	Mature neurons	Regulation of alternative pre-mRNA splicing
PV	GABAergic interneurons	Regulation of neuronal network excitability
NPY	GABAergic interneurons	Regulation of neuronal network excitability
GAD67	GABAergic interneurons	An enzyme involved in the neuronal synthesis of GABA
AChE	Cholinergic neurons	An enzyme involved in the breakdown of acetylcholine
TH	Dopaminergic neurons	An enzyme involved in the synthesis of dopamine
FJB	Degenerating neurons	Stains necrotic/injured cells
DAPI	Apoptotic cells	Stains the nucleus of apoptotic cells
TUNEL	Apoptotic cells	Stains dead cells
GFAP	Astrocytes	Responsible for the cytoarchitecture and mechanical strength
Vimentin	Radial glial/ mesenchymal cells	Responsible for the cytoarchitecture and mechanical strength
GS	Astrocytes, glutamatergic neurons	An enzyme involved in the metabolic regulation of glutamate and detoxification of ammonia by synthesis of glutamine
IBA1	Microglia	Responsible for actin bundling, membrane ruffling, cell mobility, and phagocytosis
CD4+	T-cells, microglia	Immune regulation
S100B	Astrocytes	Regulation of cell progression and differentiation, protein phosphorylation and degradation, Ca^2+^ homeostasis, energy metabolism, and innate inflammatory response
Macrophage	Microglia	Responsible for innate immunity, homeostasis, and repair of damaged tissue
TMEM119	Microglia	Unknown
CD45	Microglia	Regulates T-cell activation
CD68	Microglia	Regulates phagocytosis
DCX	Immature neurons	Involved in neuronal migration
Nestin	Neural progenitor cells	Regulation of assembly and disassembly of other IF proteins such as phosphorylated vimentin during mitosis.
NeuroD	Immature neurons	Involved in neuronal differentiation and embryonic neurogenesis
BrdU	Immature neurons	Used to detect cell proliferation
Synaptophysin	Mature neurons	Involved in the regulation of short- and long-term synaptic plasticity
MAP2	Mature neurons	Involved in the assembly, nucleation, and stabilization of microtubules
Timm	Mossy fibers	Stains zinc-containing neurons and fibers
Caspase-3	Apoptotic cells	An enzyme involved in apoptosis
Evans blue	BBB damage	Enters the brain from BBB leakage
HRP	BBB damage	An enzyme involved in catalyzing the oxidation of organic substrates using hydrogen peroxide
Endogenous plasma proteins	BBB damage	Maintenance of serum osmotic pressure
Fibrinogen	BBB damage	Involved in forming fibrin and blood clotting
SF	BBB damage	Enters the brain from BBB leakage
Dextrans	BBB damage	Enters the brain from BBB leakage
Tau	Mature neurons	Stabilization of microtubules
p-Tau	Mature neurons	Involved in the formation of neurofibrillary tangles in the brain
NfL	Mature neurons	Maintenance of axonal structure and transport
BLBP	Astrocytes	Involved in fatty acid uptake, transport, and metabolism

**Table 4:** Summary profile of primary antibodies used in lab biomarker research.

*Antibody*	*Optimal Dilution*	*Vendor*	*Host*	*Clonality*	*Marker/Target*	*Reference*
Anti-NeuN	1:1000	Chemicon, Temecula, CA, USA	Mouse	A60, monoclonal	Mature neurons	[43]
Anti-PV	1:2000	Sigma-Aldrich, St. Louis, MO, USA	Mouse	Monoclonal	GABAergic interneurons	[43,44]
Anti-NPY	1:10,000	Peninsula Labs, San Carlos, CA, USA	Rabbit	Monoclonal	Interneurons	[44]
Anti-BrdU	1:1000	Bio-Rad, Hercules, CA, USA	Rat	Monoclonal	Cells in the S phase	[45]
Anti-DCX	1:200	Santa Cruz Biotechnology, Dallas, TX, USA	Goat	Polyclonal	Newborn neurons	[44]
Anti-GFAP	1:1000	Dako North America Inc., Carpinteria, CA, USA	Rabbit	Polyclonal	Astrocytes	[43]
Anti-IBA1	1:2000	Wako Chemicals, Richmond, VA, USA	Rabbit	Polyclonal	Microglia	[43]
Anti-GAD67	1:2000	Chemicon, Temecula, CA, USA	Rabbit	Monoclonal	GABAergic interneurons	[46]
Anti-nestin	1:100	BD Biosciences, San Jose, CA, USA	Mouse	Monoclonal	Neural stem cells	[47]
Anti-vimentin	1:750	Chemicon, Temecula, CA, USA	Mouse	VIM 3B4, monoclonal	Radial glial cells	[48]
Anti-S100B	1:1	ImmunoStar Inc., Hudson, WI, USA	Rabbit	Polyclonal	Ependymal and glial cells	[47]
Anti-GS	1:400	Chemicon, Temecula, CA, USA	Mouse	Monoclonal, clone GS-6	Astrocytes	[49]
Anti-BLBP	1:100	MilliporeSigma, Burlington, MA, USA	Rabbit	Polyclonal	Astrocytes	[50]

**Table 5:** List of neural-specific markers used in neuroscience research.

*Neuron Type*	*Neural-Specific Markers*	*Full Name*	*Potential Role*	*References*
Neural Stem Cells	BRG1	Brahma-related gene 1	Regulates the oligodendrocyte progenitor’s differentiation, specification, and maturation	[53]
	MSI1	Musashi-1	Regulates target mRNA translation and promotes cell stemness, self-renewal, and tumorigenesis	[54]
	MSI2	Musashi-2	Promotes tumor proliferation, migration, and invasion	[55]
	Nestin	Nestin	Promotes cell stemness, self-renewal/proliferation, differentiation, migration, and cell-cycle regulation	[56,57]
	NCAD	N-Cadherin	Promotes tumor survival, migration, and invasion	[58]
	PAX3	Paired box protein 3	Regulates embryonic development, cell proliferation, migration, and apoptosis, and promotes cellular metastasis and invasion	[59]
	PAX6	Paired box protein 6	Promotes neural stem cell self-renewal/proliferation and neurogenesis of the CNS including the cerebral cortex	[60]
	SOX1	SRY-box transcription factor 1	Promotes stem cell maintenance and neural differentiation, and regulates embryonic development and γ-crystallin genes for developing eye lenses	[61,62]
	SOX2	SRY-box transcription factor 2	Promotes neural stem cell self-renewal/proliferation and differentiation and regulates embryonic development	[61,63]
	OTX2	Orthodenticle homeobox 2	Involved in gastrulation and brain, cerebellar, and optic nerve development	[64]
	CASPR1	Contactin-associated protein 1	Involved in astrocyte and neuron differentiation, formation, and stability of myelinated axons, and propagation of action potentials	[65]
	ASCL1	Achaete-scute family BHLH transcription factor 1	Regulates neural progenitor regulation, neuronal differentiation, and neurite outgrowth	[66]
Immature Neurons	DCX	Doublecortin	Regulates neuronal differentiation and neuronal migration by regulating the organization and stability of microtubules	[67]
	NeuroD	Neuronal differentiation 1	Promotes the development of the cerebral cortex, early retinal ganglion cells, inner ear sensory neurons, and the DG layer of the hippocampus	[68]
	TBR1	T-box brain transcription factor 1	Involved in cortical development, neuronal migration, and axonal projection	[69]
	STMN	Stathmin	Promotes neurite outgrowth in development and regeneration	[70]
	NCAM	Neural cell adhesion molecule	Regulates the adult neurogenesis, neurite outgrowth, cell migration, and fasciculation	[71,72]
	NSE	Neuron-specific enolase	Promotes neural differentiation and maturation	[73]
Mature Neurons	NeuN	Neuronal nuclear antigen	Involved in the regulation of pre-mRNA alternative splicing, neural tissue development, and regulation of adult brain functions	[74]
	Tuj1	Neuron-specific class III beta-tubulin	Involved in neuronal differentiation and neurite outgrowth	[75]
	MAP2	Microtubule-associated protein 2	Promotes the microtubule assembly and stabilization of the dendritic shape during neuronal development	[76,77]
	Tau	Tau	Promotes the microtubule assembly and stabilization by interacting with tubulin	[78]
	NFH/M/L	Human neurofilament heavy/medium/light chain	Promotes the radial growth of axons during development, the maintenance of axon caliber, and the electrical impulse transmission of axons	[79]
	Synaptophysin	Synaptophysin	Regulates the synapse formation	[80]
	PSD95	Postsynaptic density protein 95	Involved in synaptogenesis and synaptic plasticity	[81]
	GAP43	Growth-associated protein 43	Involved in presynaptic neuronal outgrowth, neuronal plasticity, axonal growth, and neurogenesis	[82]
Cholinergic Neurons	ChAT	Choline acetyltransferase	Catalyzes the synthesis of acetylcholine	[83]
	AChE	Acetylcholinesterase	Catalyzes the breakdown of acetylcholine	[84]
	VACHT	Vesicular acetylcholine transporter	Facilitates the transfer of acetylcholine from the cytoplasm into individual synaptic vesicles	[85]
Dopaminergic Neurons	TH	Tyrosine hydroxylase	Catalyzes the conversion of tyrosine to dopamine	[86]
	DBH	Dopamine beta-hydroxylase	Catalyzes the conversion of dopamine to norepinephrine	[87]
	DAT	Dopamine active transporter	Regulates the dopamine neurotransmission by terminating the action of dopamine in the synapse by reuptake	[88]
	NET	Norepinephrine transporter	Regulates the norepinephrine homeostasis via reuptake of norepinephrine into the presynaptic terminals	[89]
	Girk2	G-protein-coupled inwardly rectifying potassium channel 2	Regulates inhibitory neurotransmission and synaptic plasticity and maintains resting membrane potentials	[90,91]
	Nurr1	Nuclear receptor related-1 protein	Promotes midbrain dopamine (mDA) neural survival, development, and maturation	[92]
	Lmx1b	LIM homeobox transcription factor 1 β	Promotes mDA development, specification, and maintenance	[93]
	FoxA2	Forkhead box transcription factor 2	Promotes mDA neural survival, development, differentiation, and specification	[92,94]
	DARPP-32	Dopamine- and cAMP-regulated neuronal phosphoprotein of 32 kDa	Inhibits protein phosphatase 1	[95]
	PITX3	Paired-like homeodomain transcription factor 3 or pituitary homeobox 3	Promotes midbrain dopamine (mDA) neural survival and development	[96]
GABAergic Neurons	GABA-A receptor alpha1	GABRA1	Mediates inhibitory neurotransmission	[97]
	GABA-A receptor beta1	GABRB1	Mediates inhibitory neurotransmission	[97]
	GAD65	Glutamic acid decarboxylase 65	Catalyzes the synthesis of GABA	[98]
	GAD67	Glutamic acid decarboxylase 67	Catalyzes the synthesis of GABA	[99]
	GAT1	GABA transporter 1	Removes GABA from the synaptic cleft	[100]
	VGAT/VIAAT	Vesicular GABA transporter	Involved in GABA and glycine uptake into synaptic vesicles	[101]
Glutamatergic Neurons	GLS	Glutaminase	Synthesis of glutamate	[102]
	GS	Glutamine synthetase	Synthesis of glutamine and detoxification of glutamate and ammonia	[103]
	vGluT1	Vesicular glutamate transporter 1	Mediates the uptake of glutamate into synaptic vesicles at presynaptic nerve terminals of excitatory neural cells	[104]
	vGluT2	Vesicular glutamate transporter 2	Mediates the uptake of glutamate into synaptic vesicles at presynaptic nerve terminals of excitatory neural cells	[104]
	NMDAR1	N-methyl D-aspartate receptor subtype 1	Involved in synaptic plasticity and synaptogenesis	[105]
	NMDAR2A	N-methyl D-aspartate receptor subtype 2A	Involved in synaptic plasticity and synaptogenesis	[105]
	NMDAR2B	N-methyl D-aspartate receptor subtype 2B	Involved in synaptic plasticity and synaptogenesis	[105]
Serotonergic Neurons	5-HTT	Serotonin transporter	Transports the neurotransmitter serotonin from synapses into the presynaptic neurons	[106]
	TPH	Tryptophan hydroxylase	Catalyzes the rate-limiting reaction of biosynthesis of serotonin	[107]
	VMAT2	Vesicular monoamine transporter 2	Involved in the ATP-dependent transport of neurotransmitters into synaptic vesicles	[108]
Oligodendrocytes	OLIG1	Oligodendrocyte transcription factor 1	Promotes formation and maturation of oligodendrocytes	[109]
	OLIG2	Oligodendrocyte transcription factor 2	Promotes oligodendrocyte differentiation	[109]
	MBP	Myelin basic protein	Involved in the formation and stabilization of the myelin membranes in the CNS	[110]
	MOG	Myelin oligodendrocyte glycoprotein	Involved in the formation, maintenance of the myelin sheath, and in cell–cell communication	[111]
	MAG	Myelin-associated glycoprotein	Involved in the myelination process	[112]
	CNPase	2′,3′-Cyclic-nucleotide 3′-phosphodiesterase	Involved in the myelination process	[113]
Astrocytes	GFAP	Glial fibrillary acidic protein	Maintains the shape, strength, movement, and function of astroglial cells	[114]
	S100B	S100 calcium-binding protein B	Regulates the cytoskeletal structure and cell proliferation	[115]
	AQP4	Aquaporin-4	Maintains the ion and water homeostasis in the CNS, and mediates the astrocyte function in neuropathologies	[116]
	IGFBP3	Insulin-like growth factor-binding protein 3	Regulates IGF bioactivity, induces apoptosis, and inhibits cell growth	[117]
	ALDH1L1	Aldehyde dehydrogenase 1 family member L1	Regulates cell division and growth, involved in neural tube defects during early CNS development	[118]
	GS	Glutamine synthetase	Synthesis of glutamine and detoxification of glutamate and ammonia	[103]
	GT	Glutamine transporter	Regulates glutamate concentration	[104,119]
	Aldolase	Aldolase	Involved in glycolysis to regulate glucose homeostasis	[120]
	GLAST	Glutamate–aspartate transporter	Involved in the termination of excitatory neurotransmission of glutamate in the CNS	[121]
	Gal-3	Galectin-3	Involved in inflammation, cell adhesion, proliferation, migration, apoptosis, and tumorigenesis	[122]
	GAP43	Growth-associated protein 43	Involved in presynaptic neuronal outgrowth, neuronal plasticity, axonal growth, and neurogenesis	[82]
Microglia	CD11b	Cluster of differentiation 11b	Regulates phagocytosis, microglial migration, the production of microglial superoxide, and cell adhesion	[123]
	IBA1	Ionized calcium-binding adapter molecule 1	Regulates phagocytosis and the inflammatory response in the CNS by activating microglia	[124]
	CX3CR1	CX3C chemokine receptor 1	Regulates the inflammatory response and the synapse maturation in the CNS	[125]
	CD40	Cluster of differentiation 40	Regulates the immune and inflammatory response	[126]
	CD45	Cluster of differentiation 45	Regulates T-cell activation	[127]
	CD14	Cluster of differentiation 14	Mediates the innate immunity response to bacterial components and regulates the microglial inflammatory response	[128,129]
	CD16	Cluster of differentiation 16	Involved in phagocytosis and immune cell activation	[130]
	CD68	Cluster of differentiation 68	Involved in the inflammatory response in the CNS and regulates phagocytosis	[131]
	HLA-DR	Human leukocyte antigen-DR	Involved in cell recognition and immune modulation	[132,133]
	C1qA	Complement C1q subcomponent subunit A	Facilitates synaptic pruning/phagocytosis	[134]
	iNOS	Inducible nitric oxide synthase	Regulates systemic inflammation and sepsis	[135]
	Ferritin	Ferritin	Regulation of iron homeostasis	[136]
	Vimentin	Vimentin	Involved in cell–cell interaction, homeostasis, microglial activation, and tissue repair	[137]
	TSPO	Translocator protein	Promotes the transportation of cholesterol into mitochondria, lipid metabolism, apoptosis, proliferation, tumorigenesis, and inflammation	[138]
	P2Y12R	Purinergic receptor P2Y12	Mediates the chemotaxis process towards ADP/ATP gradients. Regulates the microglial translocation, neuronal excitability, and behavioral adaptations	[139]
	TMEM119	Transmembrane protein 119	Unknown	[140]

## Data Availability

There was no data reported in this article.
